# Double Perovskite Cobaltites Integrated in a Monolithic and Noble Metal-Free Photoelectrochemical Device for Efficient Water Splitting

**DOI:** 10.1021/acsami.1c01900

**Published:** 2021-04-27

**Authors:** Junjie Zhu, Jónína B. Guđmundsdóttir, Ragnar Strandbakke, Kevin G. Both, Thomas Aarholt, Patricia A. Carvalho, Magnus H. Sørby, Ingvild J. T. Jensen, Matylda N. Guzik, Truls Norby, Halvard Haug, Athanasios Chatzitakis

**Affiliations:** † Institute for Energy Technology (IFE), Instituttveien 18, NO-2007 Kjeller, Norway; ‡ Centre for Materials Science and Nanotechnology, Department of Chemistry, University of Oslo, FERMiO, Gaustadalléen 21, NO-0349 Oslo, Norway; § Department of Physics, University of Oslo, POB 1048 Blindern, NO-0316 Oslo, Norway; ∥ SINTEF Materials Physics, Forskningsveien 1, NO-0373 Oslo, Norway; ⊥ Department for Neutron Materials Characterization, Institutt for Energiteknikk (IFE), POB 40, NO-2027 Kjeller, Norway; # Department of Technology Systems, University of Oslo, POB 70, NO-2027 Kjeller, Norway

**Keywords:** photoelectrochemical water splitting, double perovskites, solar cells, earth abundant elements, bias-free water electrolysis, oxygen evolution reaction

## Abstract

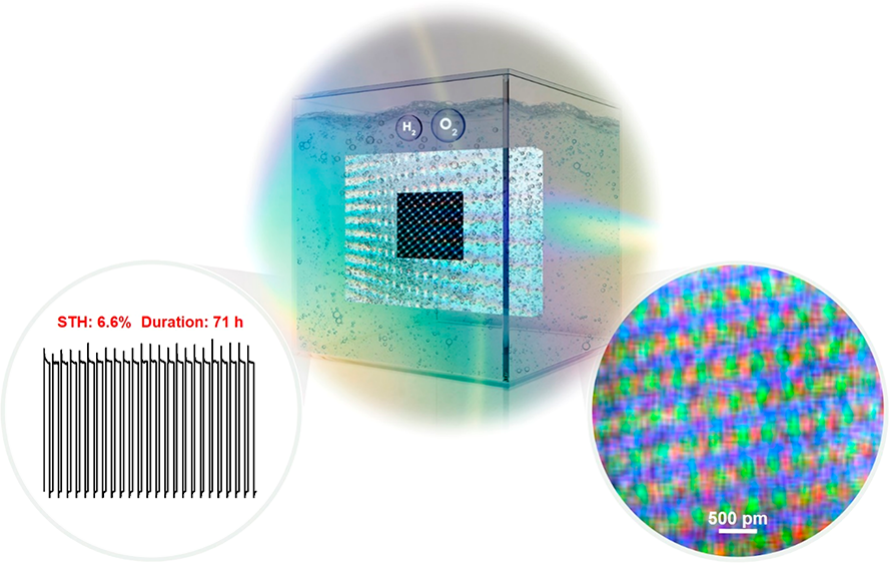

Water photoelectrolysis has the potential to produce renewable hydrogen fuel, therefore addressing the intermittent nature of sunlight. Herein, a monolithic, photovoltaic (PV)-assisted water electrolysis device of minimal engineering and of low (in the μg range) noble-metal-free catalysts loading is presented for unassisted water splitting in alkaline media. An efficient double perovskite cobaltite catalyst, originally developed for high-temperature proton-conducting ceramic electrolyzers, possesses high activity for the oxygen evolution reaction in alkaline media at room temperatures too. Ba_1–*x*_Gd_1–*y*_La_*x*+*y*_Co_2_O_6−δ_ (BGLC) is combined with a NiMo cathode, and a solar-to-hydrogen efficiency of 6.6% in 1.0 M NaOH, under 1 sun simulated illumination for 71 h, is demonstrated. This work highlights how readily available earth-abundant materials and established PV methods can achieve high performance and stable and monolithic photoelectrolysis devices with potential for full-scale applications.

## 1 Introduction

Photoelectrochemical (PEC) water splitting is categorized among the six most promising pathways for the production of renewable hydrogen gas.^1^ Solar-to-hydrogen (STH) energy conversion addresses the intermittent nature of sunlight, as well as the need for long-term energy storage and on-demand energy supply.^2^ Moreover, hydrogen is an important feedstock for the reduction of CO_2_ to hydrocarbons as well as in the fixation of N_2_ to NH_3_.^3−5^

PEC water splitting has roots back in 1972 with the pioneering work of Fujishima and Honda that spawned the modern field of artificial photosynthesis.^6^ Some more recent major breakthroughs highlighting the importance of PEC water splitting were demonstrated by Turner and Khaselev, Nocera et al., as well as van de Krol et al.^7−9^ In these works, the integration of “buried” photovoltaic junctions can provide the needed photovoltage and overpotentials for bias-free water photoelectrolysis. Immense efforts have since then been devoted to the electrolysis and photoelectrolysis of water with the key challenges still found for the complex four-electron oxygen evolution reaction (OER) and the stability of the (photoelectro)catalysts.^10−12^ Additionally, the scarcity of certain highly efficient catalyst elements, such as Ir and Ru, renders photoelectrolysis of water nonviable so far. The amounts of Ir and Ru that are needed to achieve 1 TW of hydrogen through the state-of-the-art polymer electrolyte membrane (PEM) electrolyzer represent 180 and 12 years of the current annual productions of Ir and Ru, respectively.^13^ Therefore, the development of efficient and robust catalysts based on earth-abundant elements is extremely important in order to increase the share of water electrolysis in the global hydrogen production. To that direction, oxide perovskites (ABO_3_) have shown high efficiency and stability for the OER in alkaline water electrolysis.^14−17^ The increasing interest in oxide perovskites stems from their chemical stability, as well as their structural, compositional, and electronic versatility.^18−20^

In this work, we investigate a family of double perovskite cobaltites as catalysts for the OER in alkaline media at room temperature. This work is inspired by recent advances in proton ceramic fuel cells and electrolyzers (PCFC and PCEs) that operate at elevated temperatures, i.e., 350–600 °C.^21^ The double perovskite Ba_1–*x*_Gd_1–*y*_La_*x*+*y*_Co_2_O_6−δ_ (BGLC) has a p-type electronic conductivity, which is especially important for the OER, along with a minor partial proton conductivity. BGLC (*x* = 0.5, *y* = 0.2, BGLC587) demonstrated exceptional efficiency and chemical stability as the anode electrode in PCEs at high steam pressures.^21^ Herein, we investigate the efficiency of BGLC587, BGLC82 (*x* = 0, *y* = 0.2) and BGLC37 (*x* = 0, *y* = 0.7) for the OER at room temperatures. Commercially available IrO_2_ powder is used as a reference, and our results indicate that BGLCs exhibit high intrinsic catalytic activities approaching that of commercial IrO_2_. In particular, BGLC587 shows exceptional operating stability, which is accompanied by surface amorphization. The reconstruction of the catalyst surface and formation of amorphous layers are attracting much attention as they are highly important to the overall performance of the material. It has been suggested that in the case of layered oxides and perovskites, vacant lattice oxygen sites at the surface are participating in the OER mechanism. Inequality in oxygen incorporation and evolution rates under current may be accompanied by uncoordinated cation sites, leading to cation loss and surface amorphization.^22−24^ The participation of oxygen vacancies in the OER is commonly labeled the lattice oxygen oxidation mechanism (LOM). Although oxidation of lattice oxide ions is shown to lower the electrode overpotential with respect to the adsorbate evolution mechanism (AEM), the amorphization of the surface layers indicate that evolution of lattice oxygen is faster than incorporation. Hence, the LOM has a nonfaradaic component proportional to the oxygen loss in the amorphous layer. Enhancing oxygen conductivity in the electrode bulk is suggested as a mitigation for oxygen loss^24^ but should theoretically only increase the nonfaradaic component and lead to further oxygen depletion from the electrode material. It may, however, appear as increased oxide ion conductivity is the *cause* of lowered overpotential. In reality, increasing oxygen vacancy concentration increases both oxide ion conductivity *and* OER by facilitating more surface reaction sites (vacancies). As surface amorphization is only initial and eventually comes to equilibrium with the crystalline bulk, exchange rates under current must be equal, AEM or equilibrated LOM is assumed to be dominant, and the current is all faradaic.

BGLC587 was integrated as the anode electrode in a photovoltaic (PV)-driven monolithic and “wireless” PEC cell of minimal engineering, crude handling, and minimal catalyst loadings. The PV–PEC cell based exclusively on earth abundant elements for both the OER and hydrogen evolution reaction (HER) delivered a 6.6% STH efficiency for 71 h under 1 sun simulated illumination. After the initial 71 h of laboratory operation, the PV–PEC was exposed to realistic, partially cloudy conditions with varying light intensities for 8 h. We demonstrated STH efficiencies ranging between 4.0% and 5.8% for light intensities between 0.2 and 1 sun in Oslo, Norway. Postoperation analyses highlighted the surface amorphization of BGC587 that was accompanied by Ba loss. Our work contributes to further understanding the perovskite-catalyzed OER, as well as to improving PEC water electrolysis cells for larger scale applications.

## 2 Results

**Figure 1:**
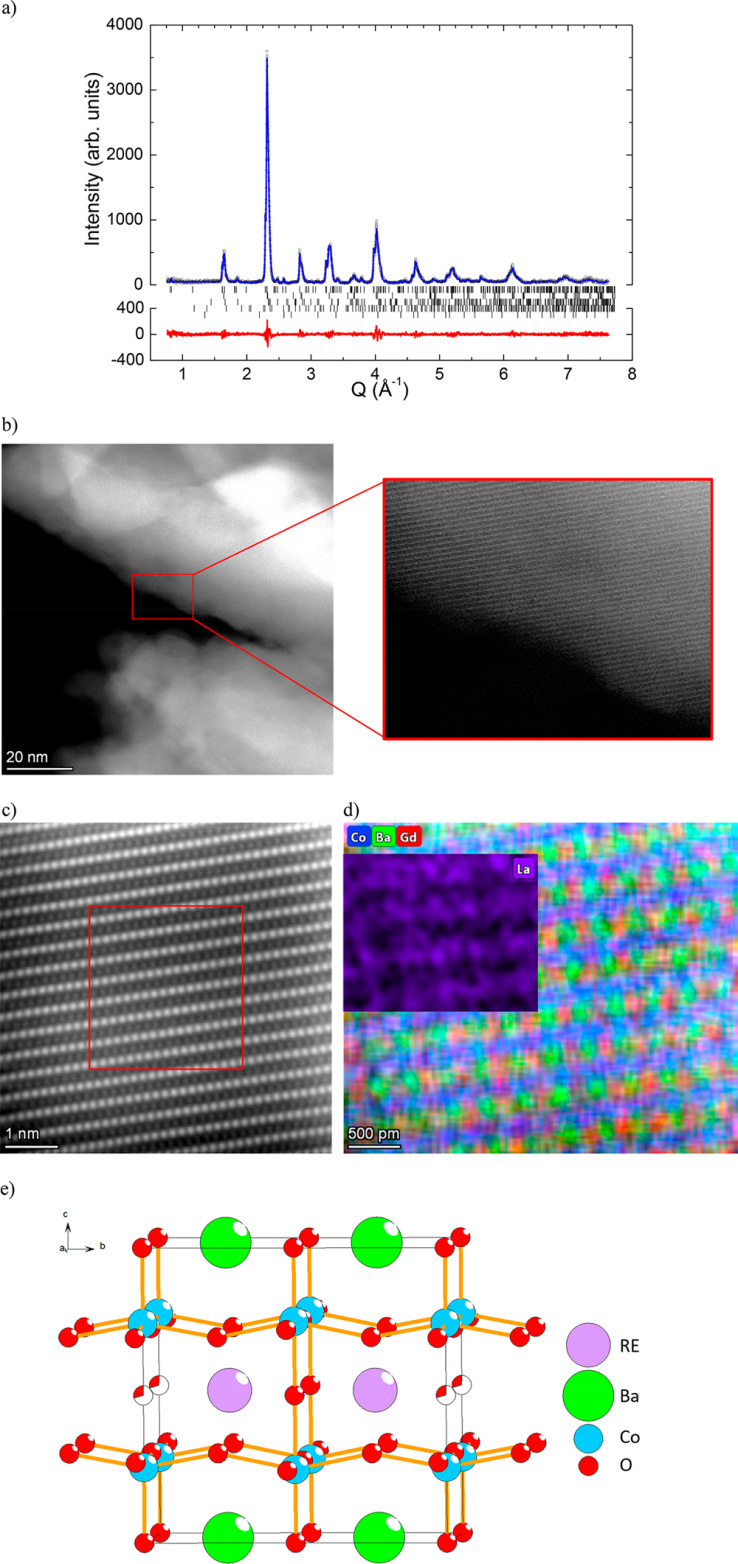
Structure and phase composition of BGLC587. (a) HR SR-PXD data with the Rietveld refinement results showing the rhombohedral LaCoO_3_-related phase (*R*3̅*c*, 50.2 wt %), orthorhombic double perovskite (*P*4/*mmm*, 21.9 wt %), Gd_0.8_La_0.2_CoO_3_ (*Pnma*, 21.3 wt %), BaCO_3_ (*Pmcn*, 4.3 wt %), and Co_2_O_3_ (*Fd*3̅*m*, 2.2 wt %). (b) STEM HAADF image of the as-received double perovskite. (c) HRSTEM HAADF image of the as-received double perovskite and (d) HREDS atomic mapping of Ba, Gd, Co, and La (inset) for the selected area in the STEM image. (e) Crystal structure of orthorhombic double perovskite. Rare earth (RE): La and Gd. Additional STEM HAADF images and STEM nanobeam scanning diffraction data on the as prepared BGLC587 are given in Figures S5 and S6.

The phase composition and structure of BGLC587, BGLC82, and BGLC37 were examined by high resolution (HR) synchrotron radiation powder X-ray diffraction (SR-PXD) for the two former and laboratory PXD for the latter. On the basis of the sample phase analysis and Rietveld refinement results, it was found that BGLC587 contains multiple crystalline phases. The major Bragg peaks are consistent with rhombohedral LaCoO_3_, a double perovskite phase, and an orthorhombic Gd_0.8_La_0.2_CoO_3_. Additional minor peaks were assigned to BaCO_3_ and Co_3_O_4_. Due to the substantial amount of LaCoO_3_ in the sample, it was assumed that the double perovskite phase is La-poor, with a composition close to orthorhombic BaGdCo_2_O_6−δ_ (space group (sg) *Pmmm*).^25^ The refined phase fractions account for 50.2(4) wt % of rhombohedral LaCoO_3_ (sg *R*3̅*c*), 21.9(4) wt % of orthorhombic BaGdCo_2_O_6−δ_ (sg *P*4/*mmm*), 21.3(3) wt % of orthorhombic Gd_0.8_La_0.2_CoO_3_ perovskite (sg *Pnma*, GdFeO_3_-type structure), 4.3(2) wt % of BaCO_3_ (sg *Pmcn*), and 2.2(1) wt % of Co_3_O_4_ (sg *Fd*3̅*m*) (Figure 1a). The refined unit cell volume of the double perovskite (229.3 Å^3^) corresponds well with that reported for BaGdCo_2_O_6−δ_ (228.6 Å^3^), thus confirming the assumption of a La-poor double perovskite formation.

To further investigate the structural characteristics of the double perovskite, scanning transmission electron microscopy (STEM) was employed for a more detailed structural analysis on the nanoscale. Figure 1b and Figure 1c show the high-angle annular dark field (HAADF) STEM images of BGLC587 with increasing magnification, revealing the high crystallinity of the material. High resolution energy dispersive X-ray spectroscopy (HREDS) atomic mapping of the cations show a structure of alternating Ba and Co layers, with Co on the B-site and La weakly distributed over the A-site in both the Ba and Gd layers (Figure 1d, inset, and additional supplementary HREDS in Figure S1). These results confirmed the solubility of La in BaGdCo_2_O_6−δ_. Such cation disorder may explain why the refined overall composition deviates somewhat from the material’s nominal composition. Specifically, the refined composition is too rich in La and too poor in Gd and Ba. This may be due to substitution of La by Gd and Ba in some of the phases. The phase described as Gd_0.8_La_0.2_CoO_3_ may for instance be more Gd-rich, with some Ba replacing La in LaCoO_3_. However, due to the similar atomic numbers of these three elements, their X-ray (synchrotron radiation) scattering contrast is too weak to confirm such a substitution by the Rietveld refinements. BGLC82 contains the same phases as BGLC587, except for Gd_0.8_La_0.2_CoO_3_, and has a higher content of the orthorhombic double perovskite phase, which accounts for 80.0(4) wt % of the sample. Its unit cell volume (229.4 Å^3^) is similar to the one observed in BGL587. The remaining phases in the sample are rhombohedral LaCoO_3_ (sg *R*3̅*c*, 10.5(4) wt %), BaCO_3_ (sg *Pmcn*, 7.5(2) wt %), and Co_3_O_4_ (sg *Fd*3̅*m*, 2.0(2) wt %) (Figure S2). The overall refined composition is La-rich and Gd-poor compared to the nominal composition, which again may be due to substitution that is difficult to detect with synchrotron X-rays. The lab-PXD data of BGLC37 did not show any additional Bragg reflections and were fitted with a single phase (a tetragonal double perovskite unit cell, Figure S3).

All the major phases are related to the perovskite structure. LaCoO_3_ and Gd_0.8_La_0.2_CoO_3_ deviate from the ideal cubic perovskite symmetry due to tilting of the CoO_6_ octahedra, which reduces the symmetries to rhombohedral and orthorhombic, respectively. The double perovskite phases have layered structures since the large size mismatch between the A-site cations induces ordering of Ba and Gd/La (see Figure 1e). The cation ordering doubles the *c*-axis, resulting in a tetragonal (*P*4/*mmm*) symmetry. This symmetry may be further lowered to orthorhombic (*Pmmm*) by oxygen vacancy ordering along the *b*-axis, resulting in an a2b2c double perovskite structure. This can be seen in the elemental mapping illustrated in Figure 1d and Figure S1 for BGLC587 and in Figure S2 for BGLC82. The larger La is more disordered between the two A-sites in the double perovskite structure. The overall morphology of the three BGLC powders and that of commercial IrO_2_, used herein as reference OER catalyst, were observed by scanning electron microscopy (SEM). The double perovskites and commercial IrO_2_ show crystal grain sizes ranging from a few hundreds of nanometers up to approximately 1 μm indicative of bulk materials (Figure S4). All powders appear with no distinct differences except that IrO_2_ has a more conical rather than cubic appearance.

The composition of BGLC587 was further investigated by XPS. The XPS investigation did not show strong indications of more than one chemical state of La, Co, and Gd present in the BGLC587 sample (Figure S7). Unlike PXD, XPS is not very sensitive to variations in long-range periodicity, as long as the local chemical environment does not change substantially. Compared to the composition found by STEM elemental mapping (Table S1), XPS detects more La and less Co (Table S2). This may suggest an La-rich outermost surface, since the XPS technique only probes a few nm into the material. Interestingly, the relative composition of Ba and Co found by XPS is in excellent agreement with the STEM results (Table S3). In the Ba 4d spectrum (Figure S7), Ba is clearly seen to be present in two different chemical states, labeled I_Ba_ and II_Ba._ The I_Ba_ component has been previously reported by Xu et al. for Ba being partially substituted by Pr in Ba_0.5_Sr_0.5_Co_0.8_Fe_0.2_O_3−δ_.^26^ This suggests that the I_Ba_ component may be a result of the partial La substitution in BaGdCo_2_O_6−δ_. Additional details can be found in Supplementary Note 1.

**Figure 2:**
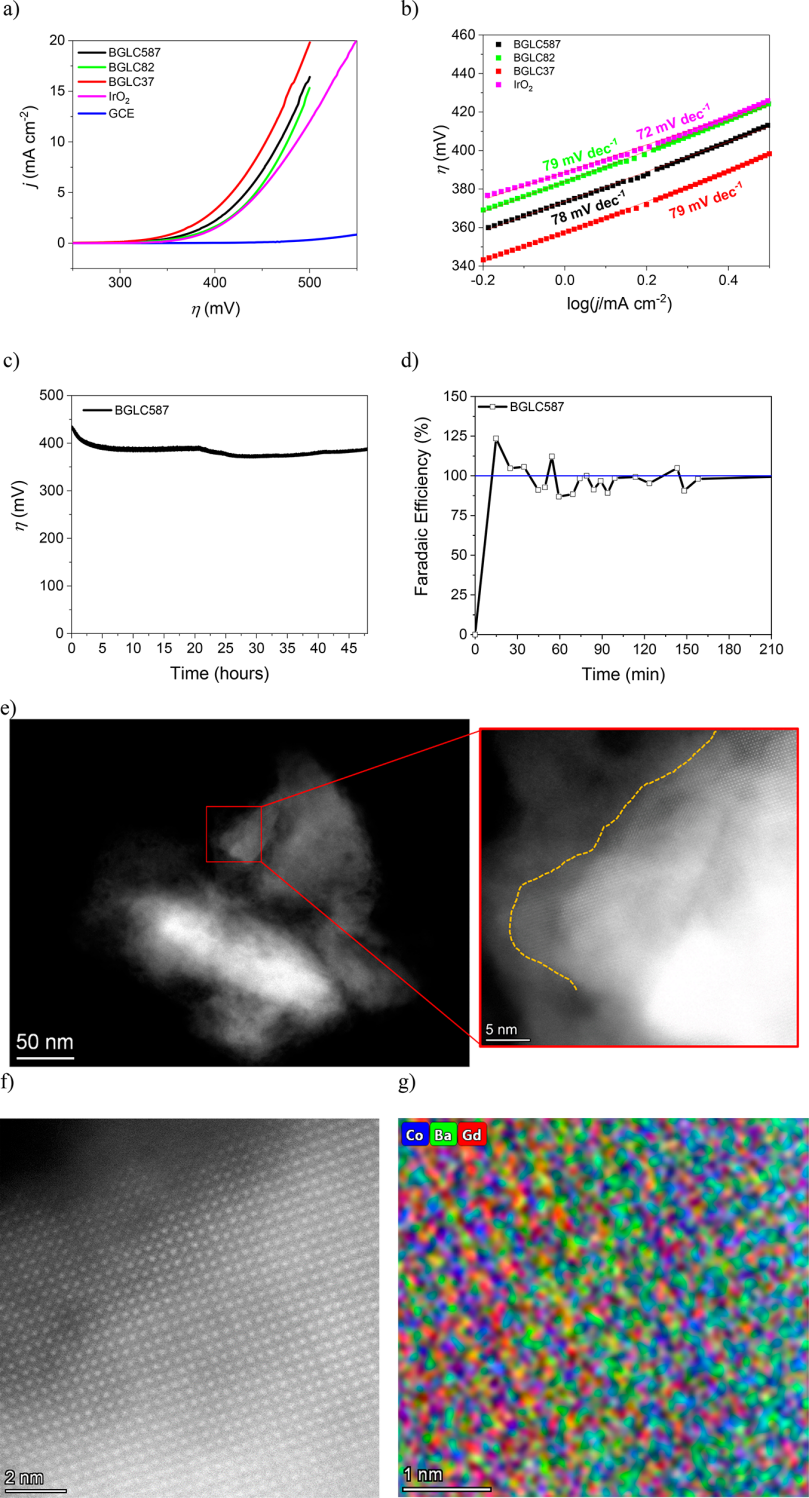
Electrochemical measurements of the double perovskite cobaltites and IrO_2_ catalysts and stability performance of BGLC587. (a) LSV curves at a scanning rate of 10 mV s^–1^ in 1 M NaOH. (b) Tafel slopes. The non-*iR*-corrected curves were used. (c) Galvanostatic stability experiment at 10 mA cm^–2^ at 1000 rpm and 0.280 mg of catalyst. Hg/HgO (1 M NaOH) was used as the reference electrode. (d) Faradaic efficiency of the BGLC587 on carbon paper loaded with 2.1 mg cm^–2^. (e) Postoperation STEM HAADF image of BGLC587. The yellow dashed line emphasizes the border of amorphous–crystalline layers. (f) HRSTEM image of the crystalline region and (g) HREDS mapping at a subsection of (f), showing postoperation A-site disorder between Gd and Ba.

The OER behavior of the three BGLC compositions against commercially available IrO_2_ in alkaline conditions is shown in the linear sweep voltammetry (LSV) of Figure 2a. The *iR* corrected curves are also given (Figure S8), but the electrolysis system is more accurately represented by the not-corrected ones.^27^ All perovskite compositions seemingly outperform the commercial IrO_2_ in both the onset overpotential (taken when *j* ≥ 0.3 mA cm^–2^)^28^ and the overpotential needed for 10 mA cm^–2^. Table 1 summarizes the overpotentials, the interfacial kinetics of the OER, and the intrinsic catalytic activities of the electrocatalysts. The perovskites all show non-*iR*-corrected Tafel slopes (Figure 2b) of around 78 mV dec^–1^, suggesting an OER mechanism with a two-electron transfer rate limiting step.^27,29^ IrO_2_ has a similar Tafel slope of 72 mV dec^–1^ in good accordance with slopes reported in the literature^30,31^ but with significantly smaller exchange current density than the perovskites. We looked into the intrinsic catalytic activities (ICA) of the BGLCs at the operating overpotential for 10 mA cm^–2^ and revealed that they are indeed inferior but approaching that of IrO_2_, especially BGLC587. The ICA of each catalyst was estimated by the *R*_ct_*C*_dl_ product, with units of Ω F that can be rearranged to (s) as the product reflects the time constant (*t*) of the studied reaction (see Table 1). We have already shown the validity of our approach,^14^ which can be used complementarily with the traditionally estimated electrochemically active surface area (ECSA) that is extracted by the capacitance in a nonfaradaic region.^27,32^ Electrochemical impedance spectroscopy (EIS) can separate capacitance and charge transfer resistance at any potential; therefore it can be applied in faradaic regions in contrast to the ECSA through cyclic voltammetry (CV). We have further validated our approach, which was applied in the nonfaradaic region, and the capacitances extracted by EIS are in good agreement with those from the ECSA (see Figures S9–S11, Tables S4–S7 and corresponding supplementary analysis). It is also noted that the capacitances get relatively lower when the oxides are in the OER region of 10 mA cm^–2^. This indicates that not all the surface area of the oxides is electrochemically active during the OER. From Table S7 it is also apparent that IrO_2_ shows the highest relative decrease in ECSA. This finding correlates well with the higher ICA found for IrO_2_ through the *R*_ct_*C*_dl_ product but also with the lowest exchange current density for the OER.

**Table 1:** Electrochemical Parameters As Estimated by the LSV and EIS Measurements of the Double Perovskites and IrO_2_

catalyst	onset at 0.3 mA cm^–2^ (mV vs NHE)	η at 10 mA cm^–2^ (mV vs NHE)	Tafel slope (mV dec^–1^), 1000 rpm	*i*_0_ at η = 0 (mA cm^–2^)	*R*_ct_*C*_dl_ – τ (s)
BGLC587	339	470	78	1.6 × 10^–5^	5.5 × 10^–4^
BGLC82	348	478	79	1.3 × 10^–5^	7.0 × 10^–4^
BGLC37	322	455	79	2.5 × 10^–5^	11 × 10^–4^
IrO_2_	359	487	72	0.4 × 10^–5^	5.1 × 10^–4^

Stability measurements were conducted under galvanostatic conditions at 10 mA cm^–2^ with a rotating disk electrode (RDE) and with the same amount of powders of 0.28 mg cm^–2^ (Figure 2c). Except for BGLC587, the rest of the catalysts were detached from the surface of the glassy carbon (GC) tip after a few hours of operation. The experiments were conducted in triplicate, and representative curves for BGLC82, BGLC37, and IrO_2_ are given in Figure S12. All overpotentials for galvanostatic operation at 10 mA cm^–2^ agree well with the ones expected by the LSV curves of Figure 2a. On the other hand, BGLC587 shows an exceptional operating stability over the course of the 48 h (Figure 2c), as well as after 300 cyclic voltammetry (CV) cycles, where 94% of the initial performance was maintained (Figure S13a). The performance of BGLC82 and BGLC37 was reduced by 24% and 17% after 300 CV cycles, respectively (Figure S13b,c). The faradaic efficiency (FE) of BGLC587 was measured under galvanostatic conditions at 10 mA cm^–2^. Although fluctuations in the oxygen production are seen due to irregular bubble release and sampling from the headspace of the electrolysis cell, the FE remained around 100% (Figure 2d) in the studied 3 h window. Therefore, all of the current can be assigned to oxygen gas generation by BGLC587. Any eventual oxygen release from the initial amorphization of the BGLC surface layers is regarded as too small to have an impact on overall efficiency. The rest of the perovskites and IrO_2_ showed a FE of around 100% for the studied 2 h window, as can be seen in Figure S12b.

After operation, BGLC587 loses the A-site ordering but maintains the crystalline perovskite structure, as can be seen in the HRSTEM image and HREDS elemental mapping for postoperation powder in Figure 2e–g. Postoperation analysis shows the expected formation of a thin (approximately 5–6 nm after 48 h of operation) amorphous layer (Figure 2e), which has been observed in several other perovskite materials operating in alkaline electrolytes.^16,33−38^ The bulk of the BGLC587 grains is still crystalline, a fact that is supported by STEM images, atomic elemental mapping (Table S1), and the nanobeam diffraction line scan from the surface and toward the bulk of the grain (Figure S14). Quantification based on XPS measurements of BGLC587 pre- and postoperation also finds a decrease in Ba content postoperation (Table S3 and Supplementary Note 1), in excellent agreement with the STEM elemental mapping. The A-site order-to-disorder transition seen from pre- to postoperation (Figure 1d and Figure 2g), as well as Ba loss (Table S1), is not seen to affect the electrochemical performance. The amorphous surface layers may be beneficial for the catalytic activity toward the OER,^22,23,33,35,36^ as before amorphization the BGLC surface is generally Co-depleted (in agreement with XPS data in Table S3) and deactivated by excess of Ba, La, and Gd. Moreover, Ba loss induces cation vacancies, to be charge-compensated by formation of oxygen vacancies or electron holes. It has previously been shown that the oxygen nonstoichiometry in BGLC587 varies little with pO_2_ at lower temperatures, and it is therefore expected that Ba loss is compensated by formation of electron holes,^39^ which further promote the OER. As mentioned previously, LOM is responsible for the surface restructuring of perovskite oxides employed as OER catalysts.^24^ The faster rate of oxygen removal compared to oxygen vacancy refilling causes the formation of uncoordinated cation sites, leading to cation dissolution and surface amorphization.^24^ Although we do not provide direct evidence, our findings corroborated well with the lattice oxygen oxidation mechanism and Ba loss, inducing electron holes, therefore facilitating the OER.

A collection of the state-of-the-art perovskites found in the literature are given in Table S8. It can be seen that BGLC587 shows high catalytic properties for the OER in alkaline media and shows promising kinetic properties (relatively low Tafel) and operating stability. Due to the higher La/Ba ratio in BGLC587 as compared to BGLC82 and BGLC37, the oxygen nonstoichiometry (and also lattice oxygen variation) is lower for the former. Still, the higher average A-site valence leaves the average Co valence lower for BGLC587 at room temperature. Co valence for BGLC587 and BGLC82 taken at 700 °C is 3.05 and 2.97, respectively,^39^ calculated to be 3.14 and 3.37 at room temperature. These values are based on oxygen nonstoichiometry obtained by thermogravimetric analysis in dry air.^39^ The inherent oxygen deficiency, mixed valence of Co, and good charge transfer characteristics between Co and oxygen supports the generally high electrocatalytic activity of BGLCs.

**Figure 3:**
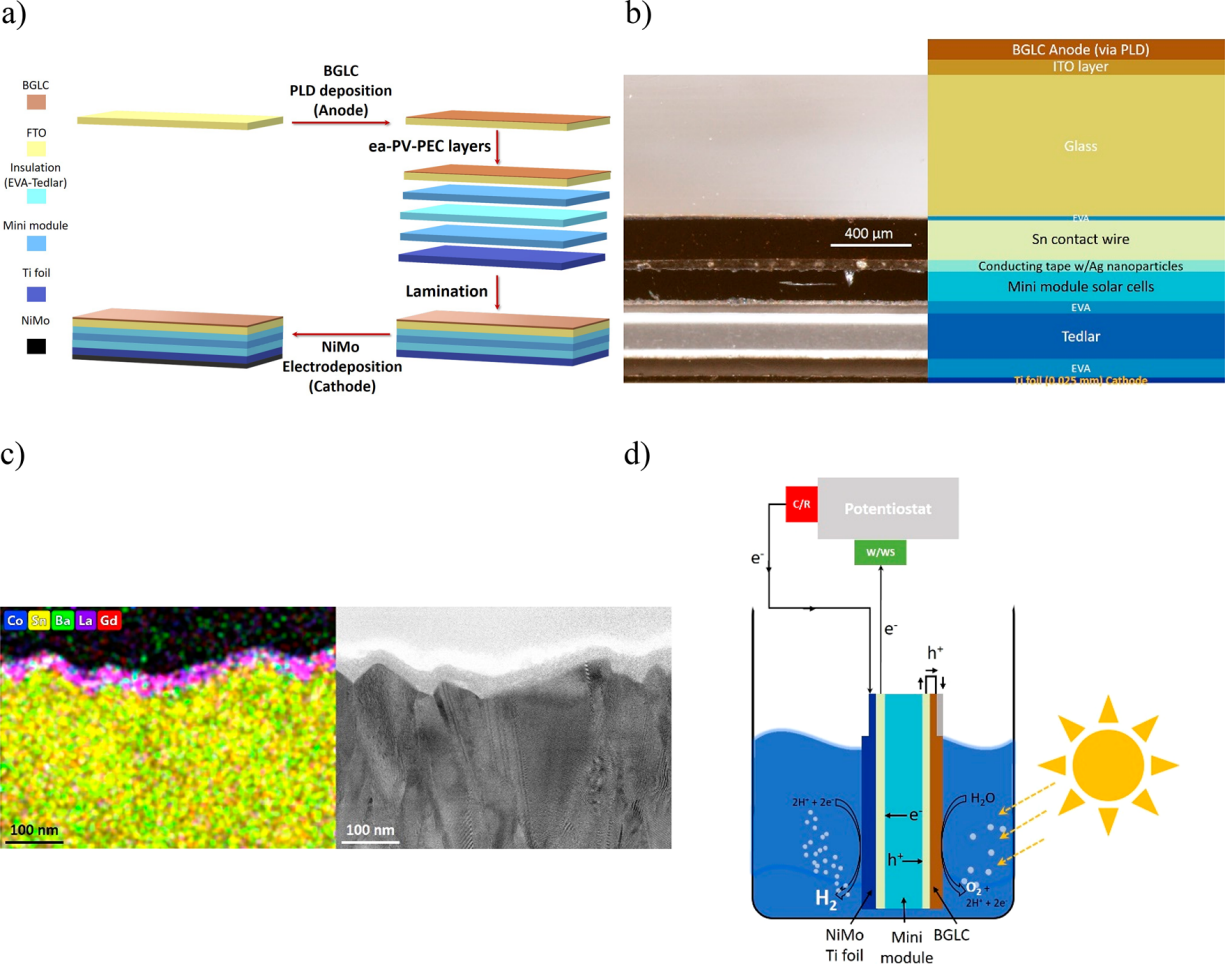
Assembly and visualization of the ea-PV–PEC device with BGLC587 as the OER catalyst and NiMo as the HER one. (a) Schematic presentation of the assembly of the ea-PV–PEC device. For simplicity, not all the layers are mentioned. The illuminated area matched the electrode area of 4 cm^2^. (b) Tomography of the ea-PV–PEC device, where the different layers in the range of a few hundreds of micrometers can be seen. It is noted that the Tedlar layer comprises three layers that are primer (top white area)–PET (middle area)–Tedlar (bottom white area). (c) Cross section STEM image of the PLD-deposited BGLC layer on FTO-coated glass (right) and the corresponding EDS analysis (left). (d) ea-PV–PEC device configuration with in series connected potentiostat. The p-terminal of the minimodule is short-circuited by the Ag ink with the anode (BGLC on FTO) and isolated by epoxy (gray part on the BGLC layer). The n-terminal of the minimodule is connected with the working/working sense (W/WS) lead of the potentiostat, while the uncoated part of the Ti foil is connected to the counter/reference (C/R) lead of the potentiostat. The flow of the electrons and holes is also mentioned. In this way we avoided having the contacts in the electrolysis solution, but the evaporating electrolyte needed compensation. The rest of the monolithic device was isolated by EVA and Tedlar sheet, which are not shown for simplicity.

A PV–PEC device was constructed with BGLC587 deposited on F-doped SnO_2_-coated glass (FTO) as the OER electrocatalyst, and a NiMo film deposited on Ti foil as the HER electrocatalyst. First, BGLC587 was deposited on the FTO by pulsed laser deposition (PLD). Then, a mini-PV module was prepared by four, series-connected solar cells (1 × 1.2 cm^2^) cut from commercial p-type monocrystalline Si passivated emitter and rear cells (PERC) with conversion efficiency of 20.5% (see connection and cross section details in Figure S15). The mini-PV module was laminated with standard ethylene vinyl acetate (EVA) as the encapsulant, cathode substrate (Ti foil) at the backside, and the BGLC587-coated FTO as the front side. After lamination, the NiMo HER catalyst was electrodeposited on the Ti foil of the whole assembly (back side of the layered structure). The assembling procedure of the whole monolithic device is schematically given in Figure 3a. A more detailed description of the assembly of the earth abundant PV–PEC (ea-PV–PEC) can be found in the experimental part, but in Figure 3b we also present a cross section of a fully functional ea-PV–PEC, where all the different layers can be seen. A lower magnification cross section image taken by an optical microscope of the full assembly can be seen in Figure S16.

PLD is a deposition technique that achieves high stoichiometry between the target and the deposited material, leading to compact films of high quality.^40,41^ Several PLD depositions under varying atmospheres were carried out in order to find the optimal conditions. The most promising results are presented in Figure S17, while deposition in O_2_ rich atmosphere gave the best performing BGLC587 layer. This resulted in a 30 nm film of BGLC587 on the FTO as it can be seen by the cross-section TEM image of Figure 3c, where a cross-section cut is prepared by the focused ion beam (FIB) technique. On the basis of Rietveld refinement of the SR-PXD data and considering phase fractions as outlined in Suppporting Information, the theoretical density of BGLC587 is 7.33 g cm^–3^, and a 30 nm layer equals to a mass of 0.023 mg cm^–2^. A brief history of the cathode development in order to reach to the electrodeposited NiMo is discussed in the Supporting Information (see Figure S18, Figure S19, Table S9 and corresponding analysis).^42^ The FE of NiMo on Ti foil was also 100% (Figure S20). In order to record the unassisted photocurrent density of the ea-PV–PEC, a potentiostat was connected in series, as described in Figure 3d. A fully standalone version can be constructed in the same way as the anode side by simply short circuiting the n-terminal with the cathode.

**Figure 4:**
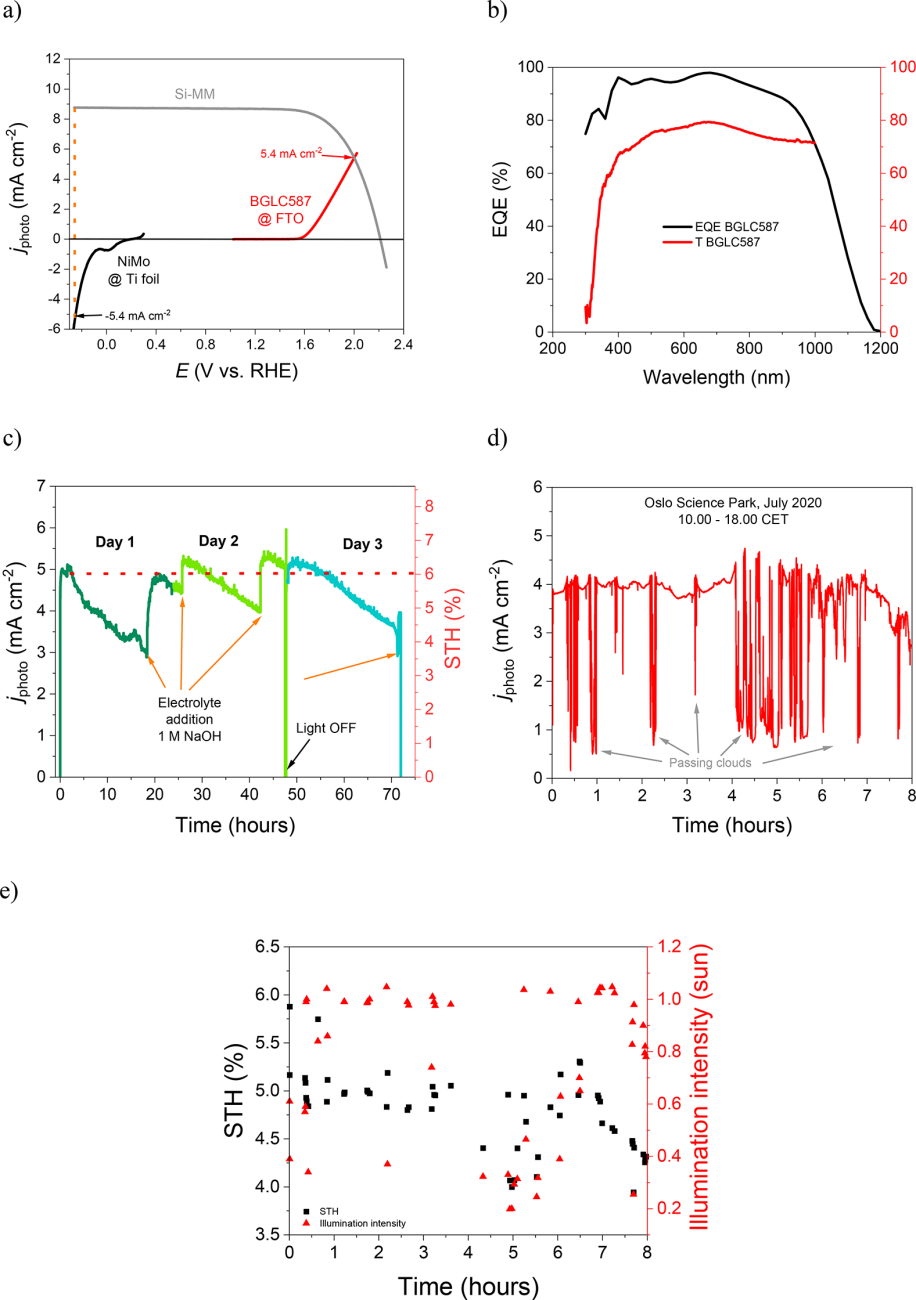
Indoor and outdoor performance of the all earth abundant PV–PEC device of 4 cm^2^. (a) Current–voltage curve of the Si-MM and current–potential curves of the BGLC587 anode on FTO (0.023 mg cm^-2^), NiMo cathode on Ti foil, and the expected photocurrent density of the ea-PV–PEC. (b) EQE and transmittance spectrum of the BGLC-coated FTO glass. (c) Indoor, bias free performance under 1 sun in 1 M NaOH. (d) Outdoor performance under varying light intensity conditions due to passing clouds. (e) Outdoor STH efficiency under varying light intensity conditions.

Before the bias-free, wireless photoelectrolysis of water, the *j*–*V* curves (the current–voltage notation of the solar cells is distinguished from the current–potential for the catalysts) of the mini-PV module (Si-MM), NiMo and BGLC587 are recorded and presented in Figure 4a. Since four crystalline Si solar cells are series connected, the *V*_oc_ of the Si-MM reaches approximately 2.3 V with a *j*_Si-MM_ of 8.76 mA cm^–2^. The expected photocurrent density is approximately 5.4 mA cm^–2^; therefore an STH of approximately 6.6% is anticipated. Figure 4b shows the optical transmittance of the glass coated with the thin BGLC587 layer and the external quantum efficiency (EQE) of the Si-MM laminated with such a glass. The BGLC587-coated glass shows over 70% of the transmittance in the visible region, where the EQE has an excellent response.

The operation of the ea-PV–PEC device under solar simulated light for more than 70 h is shown in Figure 4c. The ea-PV–PEC device under bias-free, wireless operation exhibits photocurrent densities ranging from 5.0 to 5.4 mA cm^–2^ leading to a maximum STH of 6.6%, in agreement with the projected current densities of Figure 4a. Significant fluctuation is observed though, but it is purely related to electrolyte evaporation. The geometrical parameters of the ea-PV–PEC and the possibility to connect the potentiostat in series with the cell in order to record the photocurrent density had the disadvantage that the ea-PV–PEC is submerged just enough underneath the surface of the solution (Figure 3d). It is evident that when electrolyte is added after prolonged operation, all the active surface area of the ea-PV–PEC is then fully submerged again, and the photocurrent returns to its predicted value. Moreover, a constant evaporation rate is seen by the photocurrent slopes, while the fluctuations are due to irregular gas release from the anode and cathode surfaces (the device under operation can be seen in the supplementary video 1). An additional PV–PEC device was constructed showing great consistency and reproducibility to the above mention phenomena and STH efficiency (Figure S21). We also performed light on/off cycles in the beginning and at the end of the duplicate assembly to underline that the origin of the photocurrent is purely due to the incoming illumination. An actual activity loss can be seen a little after 71 h of operation, when the ea-PV–PEC does not retrieve the photocurrent density of 5.4 mA cm^–2^ but reaches only 3.9 mA cm^–2^ after electrolyte addition (see detail in Figure S22). At this point, the whole setup was transferred outside for realistic operating conditions, and the results are given in Figure 4d and Figure 4e. First, under 1 sun intensity the STH is approximately 5%, as expected after the indoor performance loss at 71 h (Figure S22). Second, the photocurrent density fluctuates significantly due to the passing of clouds but the STH efficiency is kept constantly between 4% and 5%. This underlines the robustness of our bias-free, wireless, monolithic device under intense light/shade periods, which can be perceived as on/off cycles. The expected STH efficiencies according to the initial performance of the BGLC587 and NiMo catalysts are in a good agreement with the outdoor performance of the partially degraded ea-PV–PEC device (Figure S23). A video from the outdoor performance at 17.00 can be found in Supporting Information video 2.

The amorphization of the BGLC surface during operation in alkaline conditions is not seen to degrade the performance over time. This indicates that the surface reaction is well catalyzed by the AEM even when Co oxide is suspended in an amorphous matrix of Ba, Gd, and La oxides or hydroxides. But due to high partial oxide ion conductivity and since the total BGLC layer is only 30 nm thick, the complete amorphization after 71 h (Figure S24) leads to an irreversible performance degradation, emphasizing the significance of crystalline BGLC on cell performance. SEM observation of the NiMo film also indicates degradation of the HER catalyst. The EDS analysis shows an increase in the Ni:Mo ratio from 8.5 to 10.6, as well as an increase in the detection of the Ti substrate, indicating a partial loss of the NiMo film during the operation of the cell (Figure S19 and Table S9). The Si-MM was intact and the *j*–*V* characteristics were not affected (data not shown).

## 3 Discussion

**Figure 5:**
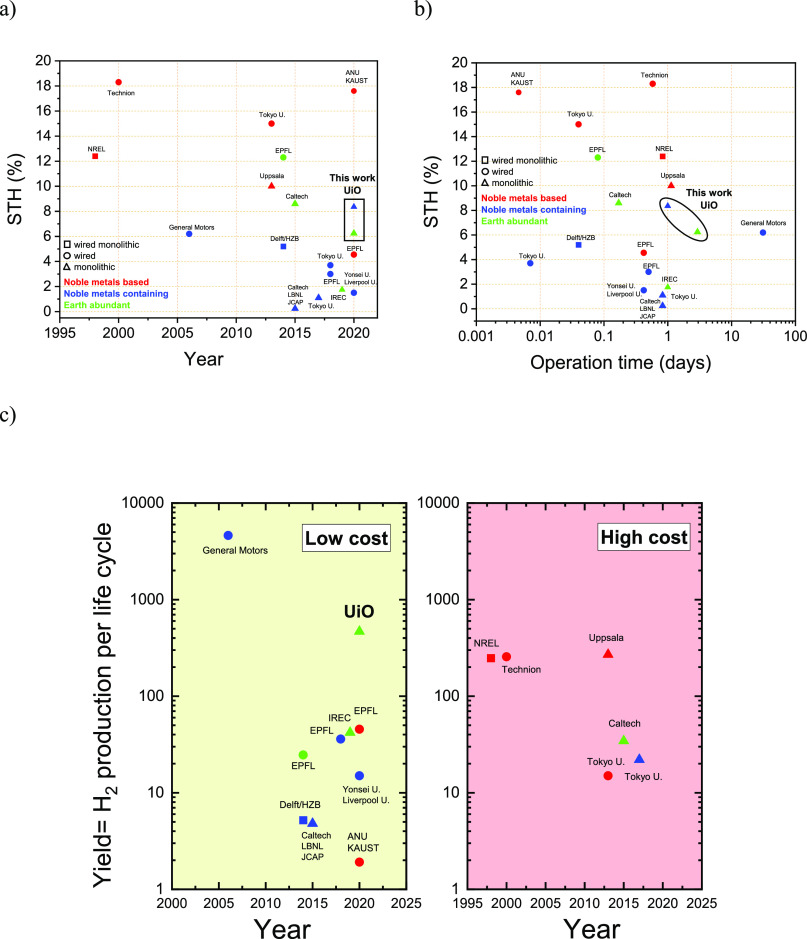
STH efficiency and stability benchmarks for a wide range of bias-free photoelectrolysers. (a) Some of the most important works since the pioneering work of Khaselev and Turner’s. (b) Their operating durations. All works are summarized in Table S10. “Wired monolithic” refers to photoelectrolyzers where at least one electrode contains a “buried” PV junction and it is wired to the other electrode. “Wired” refers to purely PEC-based devices that are not assisted by “buried” PV junctions and PV-assisted electrolyzers. Finally, “monolithic” refers to purely tandem designs with no external wires. The majority of the devices used, either for the anode or cathode or for both electrodes, noble metal-based catalysts. The all-earth-abundant devices are denoted by green triangles. (c) Apparent H_2_ production per life cycle of each solar water splitting system taking into account that in the reported operating times the systems did not degrade. The low-cost category includes systems that use Si-based solar cells, perovskites (although their market price is currently unknown, we regarded them as low-cost photoabsorbers), and photoelectrodes based on common semiconductors (BiVO_4_, Cu_2_O, etc.). The high-cost category includes III–V- and CIGS-based solar cells.

Figure 5a and Figure 5b highlight the most important works in the field of unassisted water electrolysis in terms of both STH and stability of the presented devices since the ground-breaking work of Khaselev and Turner in 1998.^7^ Our monolithic, wireless water splitting device has the second highest STH efficiency reported so far after the device by Verlage and co-workers in 2015.^43^ They achieved an STH of 8.6% with a monolithic, “wireless” water splitting device of 1 cm^2^. The device was based on earth-abundant elements exclusively, but it had limited stability reporting a 10% degradation after 4 h of laboratory conditions. Our STH of 6.6% is lower by 23%, but it showed no degradation in electrochemical performance for 71 h operation in laboratory conditions. Although our device needs 4 times more area to reach this high STH efficiency compared to Verlage et al., the cost of our Si solar cell used is negligible compared to a GaAs-based device (retail price of a triple-junction GaAs solar cell is ∼$60 for 1 cm^2^, while a piece of 6 in. Si based high efficiency solar cell (243 cm^2^) costs less than $1). The advantages of series-connected solar cells in terms of complexity when compared with tandem solar cells are also highlighted by Jacobsson et al.^44^ A chemical degradation could however be seen, as the amorphous surface layer gradually propagated throughout the thickness of the 30 nm electrode film. The loss of the crystalline backbone of the perovskite leads to additional charge transfer losses, a fact that poses important scientific challenges. Operando investigations of such degradation phenomena could also provide important insights on the role of each phase. Such in-depth studies should be further pursued.

Additionally, we have constructed a bias-free, wireless monolithic device with highly active IrO_2_ nanoparticles electrodeposited as the anode electrode^45^ instead of BGLC587. The electrodeposition of IrO_2_ was carried out after the lamination of the 4 cell connected Si-MM and the electrodeposition of NiMo in the cathode. The device reached an STH of 8.4% that is almost as high as the monolithic device by Verlage et al., but the performance was reduced by 11% in the course of 24 h. The performance loss was attributed to the IrO_2_ dissolution, as it could be re-electrodeposited and the performance be retrieved (Supplementary Note 2).

The high performance of our ea-PV–PEC is also underlined by the apparent H_2_ yield given in Figure 5c. The yield of each system was calculated by multiplying the reported STH with the operating time in hours. The units could be regarded as yield of hydrogen in hours of operation showcasing the accumulation of fuel over the course of a stable cycle of each device. The two categories are roughly defined by the cost of the photoabsorbers only, not taking into account the cost of noble metals used as catalysts. Although the system developed by General Motors back in 2006 is the only one exceeding our ea-PV–PEC device,^46^ it was partially based on noble metals and was not a monolithic one.

## 4 Conclusions

We have demonstrated a state-of-the-art monolithic solar water splitting device with minimum engineering and low loadings of novel and earth abundant catalysts. The anode of our ea-PV–PEC device was based on a new class of double perovskite cobaltites for the OER of the general formula Ba_1–*x*_Gd_1–*y*_La_*x*+*y*_Co_2_O_6−δ_ (BGLC) and showed high catalytic activity, reporting a 6.6% STH under 71 h of continuous illumination. HRSTEM HAADF imaging of BGLC587, which was the best performing among the presented double perovskites, revealed an A-site order-to-disorder transition from pre- to postoperation that did not affect the catalytic activity for the OER. On the contrary, the surface amorphization assisted in exposing the Co species, thus activating the AEM on BGLC, although a crystalline bulk backbone is necessary in retaining charge transfer (i.e., electron holes). The loss of Ba is predicted to lead to further Co oxidation by formation of electron holes to charge-compensate the cation vacancies. A partial amorphization of BGLC587 is most probably related to lower incorporation than evolution rates in the lattice oxygen oxidation mechanism under current. Due to fast oxygen transport in BGLC587, oxygen vacancies at the crystalline surface can be refilled and the LOM may dominate over AEM until a critical thickness of the amorphous layer is reached. Given the thin electrode layer of the ea-PV–PEC, full amorphization of the 30 nm thick BGLC film led to irreversible activity loss after 71 h of operation. On the other hand, the structure rearrangement and surface amorphization of OER catalysts are an open topic and a current knowledge gap that will assist in the development of catalyst materials of immense activity.^23^

Further improvements in lowering the overpotentials for the OER and HER will lead to even higher STH efficiencies and prolonged operating times by using commercially available and affordable Si-based solar cells. It is highlighted that the currently used lamination process shows potential for long-term operation of such PV–PEC devices in strongly alkaline media. The integration of high voltage, high current solar cells (e.g., affordable III–V semiconductors) can also be facile and can further boost the performance and also reduce land usage and costs. Our results are based on simple, scalable processes as well as on readily available materials that widen up research possibilities, bridging the gap toward commercially viable solar water electrolysis.

## 5 Experimental Section

### 5.1 BGLC Anode Electrode Preparation

BGLC powders (BGLC587, BGLC37, and BGLC82) were purchased from Marion Technologies (F) and were used as received. The synthesis can be performed by the sol–gel citrate method as described in Supplementary Note 3. BGLC587 was deposited on FTO coated glass by pulsed laser deposition (PLD, Surface-Tec system, laser Coherent COMPexPro 205F, KrF, wavelength of 248 nm). The films were deposited at 100 °C in an oxygen-rich environment (0.01 mbar) with 4.5 J cm^-2^. The repetition rate was 5 Hz, and the distance between target and substrate was 9 cm.

### 5.2 NiMo Cathode Preparation

NiMo was electrodeposited on Ti foil (thickness 0.025 mm, Goodfellow, ≥99.6+%) in a two-electrode cell according to the procedure described by Fan et al.^47^ with certain modifications. The molarities of NiSO_4_·6H_2_O (Sigma-Aldrich), Na_2_MoO_4_·2H_2_O (Sigma-Aldrich), and Na_3_C_6_H_5_O_7_·2H_2_O (Sigma-Aldrich) were kept the same, but the electrodeposition was carried out stepwise under potentiostatic control at 2.85 V. The Ti foil substrate served as the cathode electrode, and a Ni foam was used as sacrificial anode with the nominal area for both electrodes being 4 cm^2^. Four electrodeposition steps are discerned with the main difference that in the two first steps the solution was not stirred, while in the following two steps the solution was stirred. Each step was applied successively and lasted for 60 s, and the current ranged between 33 and 40 mA cm^–2^. This procedure gave visually the most homogeneous coatings, as well as optimized performance of the NiMo cathodes.

### 5.3 IrO_2_ on FTO Anode Preparation

Highly active IrO_2_ on FTO glass (either stand alone or on the assembled mini-PV module) was deposited by electrodeposition adapted to the procedures described by Zhao et al.^45^ The electrodeposition was carried out in a three-electrode configuration with the FTO glass, standard calomel electrode (SCE), and a Pt mesh as the working, reference, and counter electrodes, respectively. After a brief optimization of the deposition time (see Figure S17) it was found that 3.5 h at +1.45 V vs SCE produced the best performing IrO_2_ film, which did not compromise the transparency of the FTO glass (see EQE and transmittance spectrum in Figure S29).

### 5.4 Materials Characterization

HR SR-PXD data for BGCL587 and BGLC82 were collected at BM31 of Swiss-Norwegian Beamlines, at ESRF in Grenoble (France). Powders were sealed in a boron glass capillary with an internal diameter *d* = 0.3 mm and measured over an angular range of 1–35° 2θ with a step size of 0.006° 2θ with 6 scintillation detectors, each fitted with a Si analyzer crystal in front. The wavelength (λ = 0.502 18 Å) was calibrated using Si as a standard material. Laboratory PXD data for BGLC37 were collected in a Bragg–Brentano geometry with a Bruker-AXS D8 Discovery diffractometer, equipped with a LynxEye 1D detector, and CuKα_1 radiation was selected by a Ge (111) monochromator. The angular range used was 10–90° 2θ, with the step size of 0.02° 2θ. Phase identifications from (SR-)PXD data were performed by search–match with the PDF4-2020 database embedded in the Bruker EVA software. Structure refinements and quantitative phase analysis were performed by the Rietveld method in the TOPAS Academic version 5.^48^ The Bragg peak profiles were described by Thompson-Cox-Hastings pseudo-Voigt functions,^48^ and the backgrounds were fitted with Chebychev polynomials.

STEM work was performed with a Titan G2 60-300 instrument, operated at 300 kV with 80 pA beam current and 0.08 nm of nominal spatial resolution. The samples were investigated using data collected by annular bright-field (ABF), low-angle annular dark-field (ADF), and high-angle annular dark-field (HAADF) detectors. Chemical information was obtained by X-ray energy dispersive spectroscopy (EDS) with a Bruker SuperX EDS system, comprising four silicon drift detectors. Convergence angle was set to 21 mrad for EDS and high-resolution and to 1.75 mrad for nanobeam scanning diffraction. STEM sample preparation was performed by focused ion beam (FIB) with Ga+ ions accelerated at 30 kV using a Thermofisher Helios multibeam system.

XPS was performed on a Kratos Axis Ultra DLD spectroscope with monochromated Al Kα X-rays (*h*ν = 1486.6 eV). Survey spectra were obtained using pass energy (PE) of 160 eV and step size of 1 eV, while PE of 20 eV and step size of 0.1 eV were used for high resolution spectra.

The SEM images were obtained with a Hitachi SU8200 ultrahigh resolution cold-field emission scanning electron microscope equipped with a secondary electron (SE) detector under an acceleration voltage of 2.0 kV.

An operating PV–PEC device was embedded in epoxy, and the cross section was observed by an optical microscope after standard metallographic preparation.

### 5.5 Rotating Disk Electrode Electrochemical Measurements

The electrochemical experiments were performed in 1 M NaOH solution in a three-electrode setup provided by Gamry. An RDE with a GC tip (RDE710 rotating electrode, Gamry Instruments) was used as the working electrode, and SCE was used as the reference and a graphite rod as the counter electrodes, respectively. The standard potential of the SCE was measured and calibrated against a reference SCE, as well as a reference Ag/AgCl (3 M KOH) after each experiment. The RDE tip was coated by the perovskite and IrO_2_ (Sigma-Aldrich, CAS 12030-49-8) powders according to the procedure suggested by Zhu et al.^34^ The catalyst ink was prepared by adding 10 mg of powder in 1 mL of ethanol and 100 μL of Nafion 5 wt % solution. The powder inks were sonicated for a few hours until a homogeneous suspension was obtained. The inks were drop-casted on the GC tip (0.196 cm^2^) by applying 6 μL of ink and allowed to dry in air. This procedure resulted in a loading of approximately 0.280 mg cm^–2^ of the electrocatalyst. For long-term stability experiments, the same amount of powder was loaded by drop-casting on the RDE. In this case, Hg/HgO (1 M NaOH) was used as the reference electrode as SCE is not appropriate. The standard potential of the Hg/HgO (1 M NaOH) reference electrode was found according to our reference SCE. All the electrochemical measurements, such as cyclic voltammetry (CV), linear sweep voltammetry (LSV), electrochemical impedance spectroscopy (EIS), chronoamperometry (CA), and open circuit potential (OCP), were performed with a Gamry Reference 3000 potentiostat/galvanostat/ZRA. All overpotentials are given against the normal hydrogen electrode (NHE) taking into account that water electrolysis takes place thermodynamically at 1.23 V vs NHE. Potentials were corrected vs NHE according to the Nernst equation:
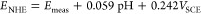
and
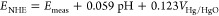


### 5.6 PV–PEC Device Assembling and Performance Testing Indoor and Outdoor

The mini-PV module contains four pieces of monocrystalline Si solar cells (1 × 1.2 cm^2^ each) with shingled interconnection. The interconnection was realized by Ag nanoparticles containing double-sided conductive tape (3M, 220-9928). The interconnection overlap between the solar cells is 2 mm, resulting in an illumination area of 4 cm^2^ indeed. The solar ribbon was attached to p- and n-terminal of the minimodule with the Ag-containing double-sided tape. The layered structure with the BGLC587-coated FTO, EVA, interconnected solar cells, EVA, Tedlar sheet, EVA, and Ti foil (0.025 mm) was laminated at 150 °C with standard lamination process. According to the manufacturer, the Tedlar DyMat CTE white sheet comprises a top primer layer, which is adhered to a PET (hydrolysis resistant, electrical grade) midlayer that is adhered to the Tedlar bottom layer.

After the lamination of the mini-PV module, the deposition of the HER catalyst took place. The electrodeposition of NiMo was performed on the Ti-foil as described previously and directly on the assembled layered structure. After the deposition of the cathode catalyst on the monolithic device, the ribbon from the n-terminal of the mini-PV module was connected to the cathode electrode, while the ribbon from the p-terminal was connected to the anode with Ag ink (Loctite). The ink was applied with brush, and it was cured in air after 2 h. Two layers of the Ag ink were necessary such that they were finally isolated by epoxy resin (Huntsman Araldite 2000). In the end, the circumference of the device was covered by epoxy in order to further protect the potential shunting due to the liquid.

The STH and PEC performance of the monolithic, wireless PV–PEC device was measured in 1 M NaOH under illumination of an AM 1.5G solar simulator (Newport Oriel LCS-100). The light intensity was regularly calibrated by a reference solar cell (Newport 91150V-KG5). The photocurrent density was recorded by a Gamry Reference 3000 potentiostat, which was connected in series with the monolithic device. For this purpose, an alternative connection was used in which the cathode of the monolithic device was connected to the reference/counter leads of the potentiostat, while the n-terminal was connected to the working/sense leads. The potentiostat was set to 0 V vs *E*_ref_, and the connections are schematically given in Figure 3d. This series resistance was not compensated in our results.

For the indoor laboratory experiments, the monolithic, “wireless” device was inserted without any further modification in an approximately 300 mL solution of 1 M NaOH in a glass reactor of approximately 1 L that was equipped with a 5 cm × 5 cm flat quartz window. For the outdoor experiments under realistic conditions, the whole setup, including the monocrystalline Si PV reference cell, was placed side by side on a portable table and taken to the terrace on the fifth floor of the Oslo Science Park. The proper angle of approximately 45° was adjusted by measuring the light intensity inside the cell, in front of the quartz window before the experiment was initiated. The angle was kept constant throughout the experiment, and the sun was tracked manually. An 8 h long experiment from 10.00 to 18.00 CET was performed in July 2020 at the location WPR8+RG Oslo. The sunlight intensity and temperature of the monocrystalline Si PV reference cell were regularly recorded, while the photocurrent density was recorded by the Gamry potentiostat as in the indoor experiments.

### 5.7 Gas Quantification

The O_2_ and H_2_ gases were measured with an Agilent 3000A Micro GC, and the gas samples were collected automatically every 5 min from an 80 mL headspace. N_2_ gas was used to remove the dissolved O_2_ gas from the solution, as well as the air in the headspace. The removal of O_2_ from the electrolysis cell (total volume of 160 mL) was also monitored before the initiation of the faradaic efficiency (FE) experiments. The working electrodes for the FE measurements were carbon paper loaded with around 5 mg of each catalyst powder, while a Pt mesh was used as the counter electrode. The inks consisted of 160 mg of catalyst powder, 3.4 mL of water, 1 mL of isopropanol, and 40 μL of Nafion, 5 wt %.^49^

## Supporting Information Available

Additional figures and tables of results and Supplementary Notes 1–3 on XPS analysis, performance characteristics, and synthesis information (PDF)
Video 1 showing operation of the ea-PV-PEC device (MP4)
Video 2 showing outdoor performance of the ea-PV-PEC device (MP4)
