# Supplementary material for: Double Perovskite Cobaltites Integrated in a Monolithic and Noble Metal-Free Photoelectrochemical Device for Efficient Water Splitting
Source: ACS Appl Mater Interfaces. 2021 Apr 27;13(17):20313–25. doi: 10.1021/acsami.1c01900 (PMC8289171; doi:10.1021/acsami.1c01900)
Supplement: Supplementary file 1 — am1c01900_si_001.pdf [file am1c01900_si_001.pdf]

Supporting Information (SI)

Double perovskite cobaltites integrated in a monolithic and noble metal-free photoelectrochemical device for efficient water splitting

Authors

Junjie Zhu,[1] Jónína B. Guðmundsdóttir,[2] Ragnar Strandbakke,[2] Kevin G. Both,[2] Thomas Aarholt,[3] Patricia A. Carvalho,[4] Magnus H. Sørby,[5] Ingvild J. T. Jensen,[4] Matylda N. Guzik,[6] Truls Norby,[2] Halvard Haug,[1] Athanasios Chatzitakis[2*]

Affiliations

[1] Institute for Energy Technology (IFE), Instituttveien 18, NO-2007 Kjeller, Norway

[2] Centre for Materials Science and Nanotechnology, Department of Chemistry, University of Oslo, FERMiO, Gaustadalléen 21, NO-0349 Oslo, Norway

[3] Department of Physics, University of Oslo, POB 1048 Blindern, NO-0316 Oslo, Norway

[4] SINTEF Materials Physics, Forskningsveien 1, NO-0373 Oslo, Norway

[5] Institutt for Energiteknikk (IFE), Department for Neutron Materials Characterization, POB 40, NO-2027 Kjeller, Norway

[6] Department of Technology Systems, University of Oslo, POB 70, NO-2027 Kjeller, Norway

[*]Corresponding author: a.e.chatzitakis@smn.uio.no

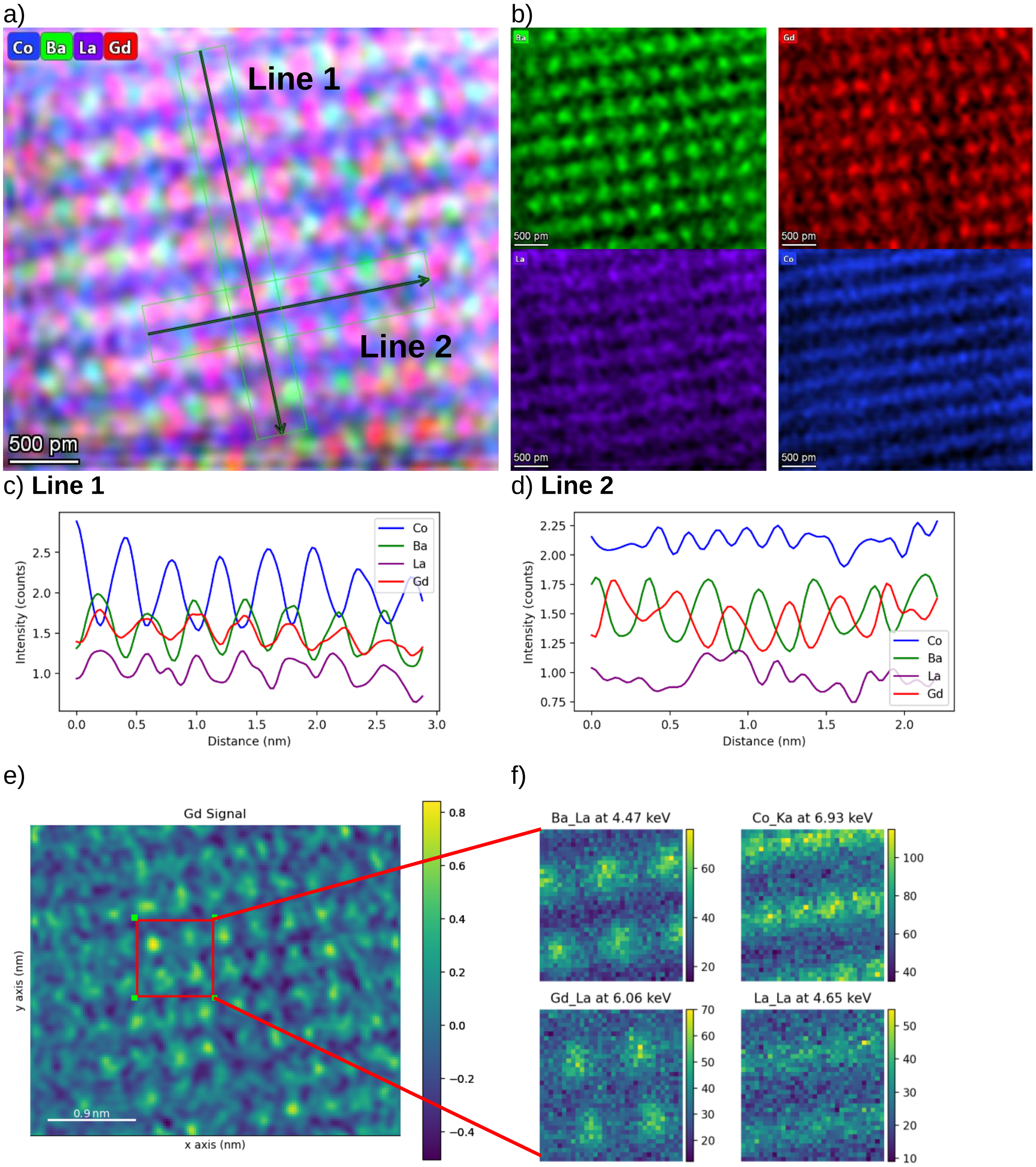

Figure S1: STEM-EDS maps showing the presence of La, Ba, Gd and Co. Composite image showing position of elements and line profiles (a). Individual maps from a) (b). Line profiles along the 010 and 001 direction showing Co on alternating positions to the other elements (c), and Ba and Gd on alternating positions with Co and La spread out across the columns (d). Gd raw signal showing positions of Gd columns used for template matching of the motif highlighted in red (e). The motif stacked across the image and quantified to improve signal-to-noise-ratio showing stacked counts, in order to emphasise the positions of the elements including the solubility of La (f).

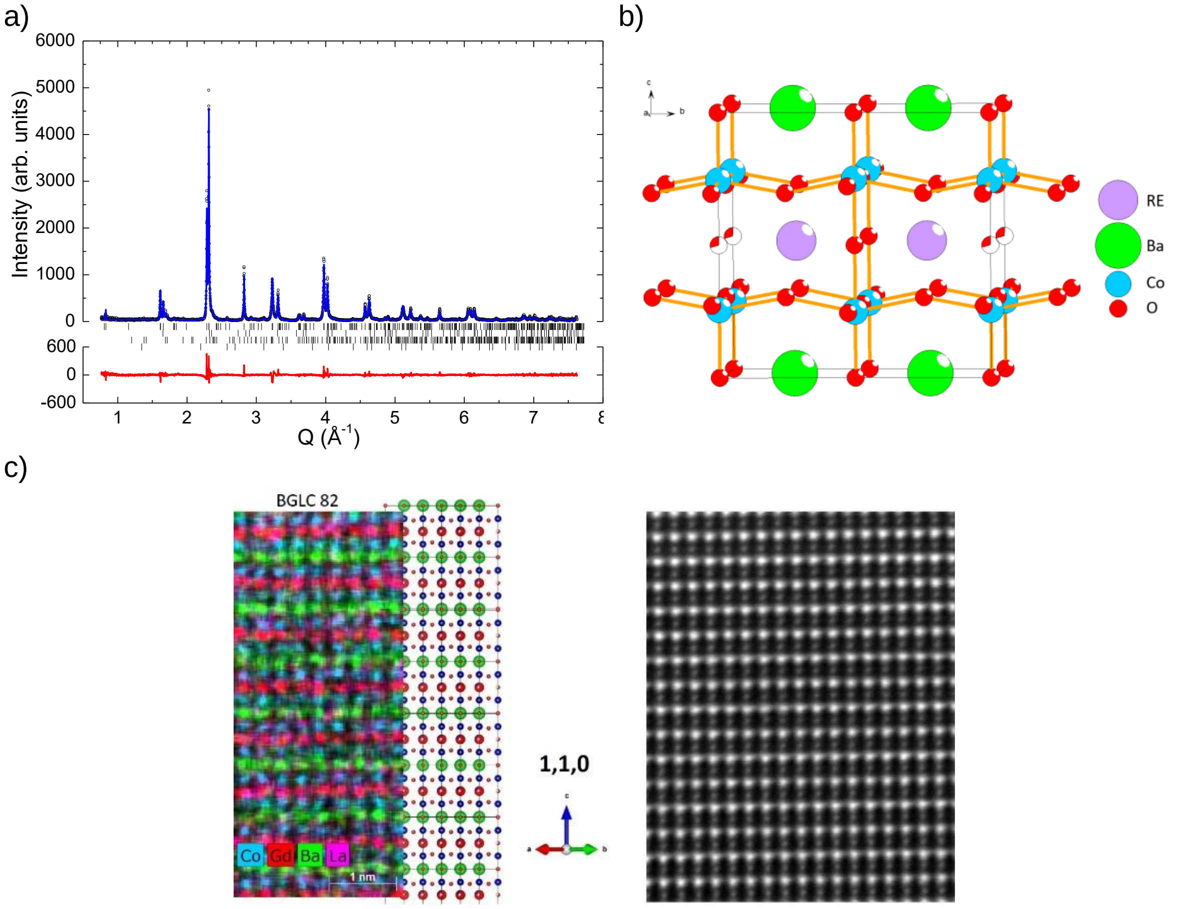

Figure S2: SR-PXD data of BGLC82 and the Rietveld refinement results with the main phase (*Pmmm*, 80.0 wt%), and secondary phases Co_3O_4 (2.0 wt%), $BaCO_3$ (7.5 wt%), and $LaCoO_{2.9}$ (10.5 wt%) (a). *Pmmm* structure of BGLC82. Rare Earth (RE): La and Gd (b). STEM EDS mapping and HAADF image of BGLC82 in as-received condition (c).

a)

b)

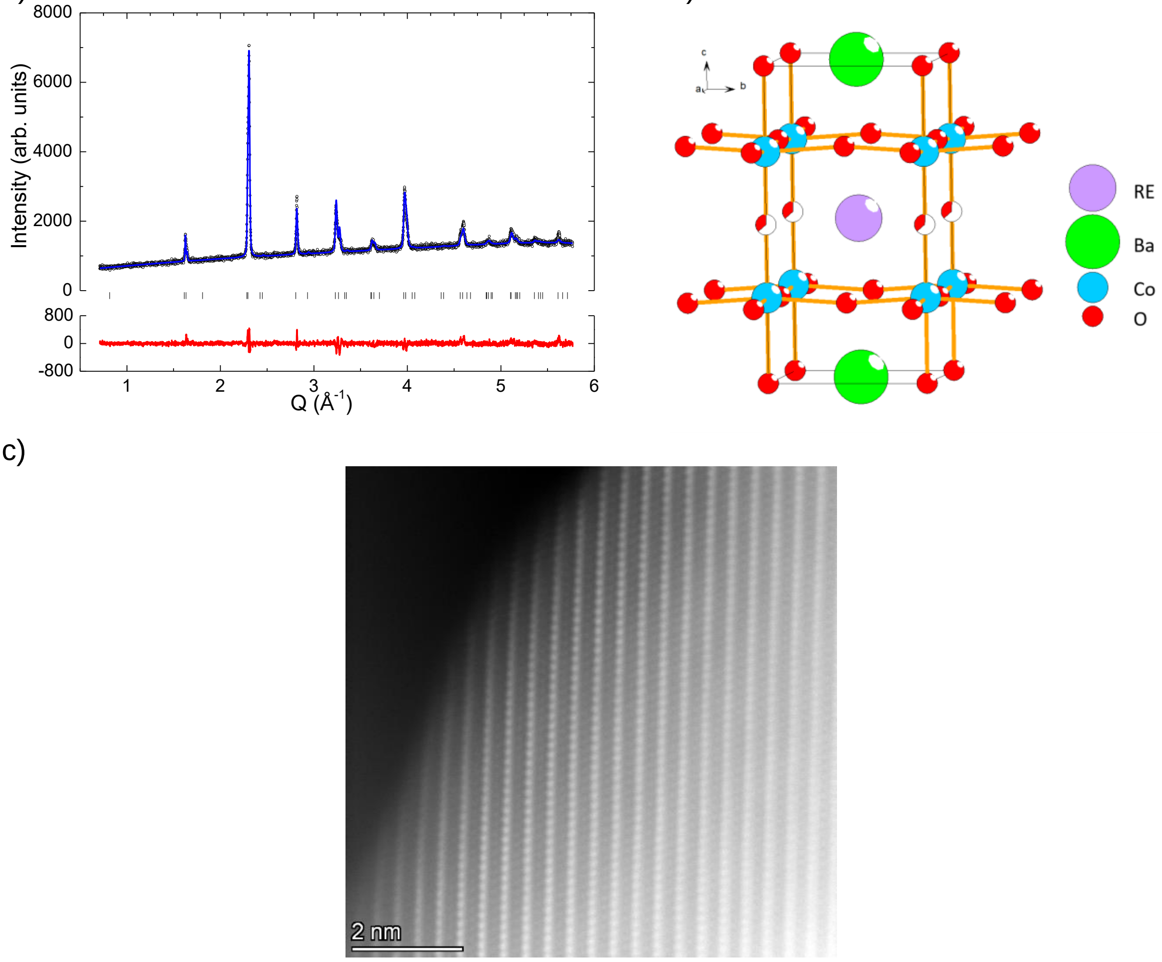

c)

Figure S3: SR-PXD diffractogram of BGLC37 with Rietveld refinement (a). *P4/mmm* structure of BGLC37. Rare Earth (RE): La and Gd (b). HR STEM image of hydrated BGLC37 (c).

a)

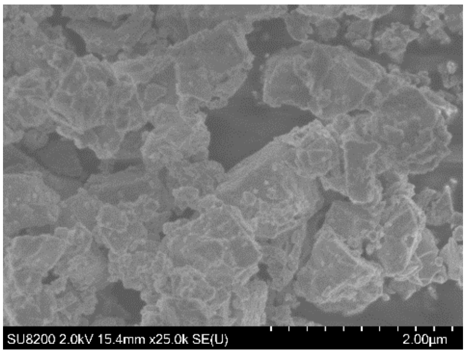

b)

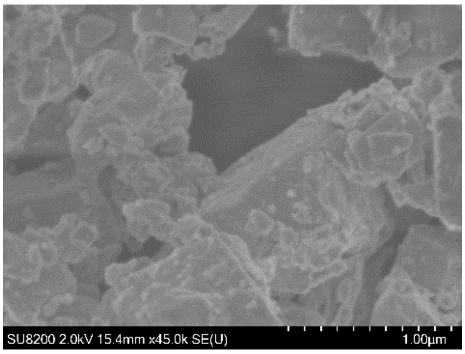

c)

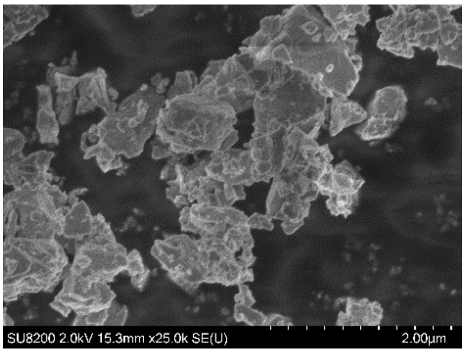

d)

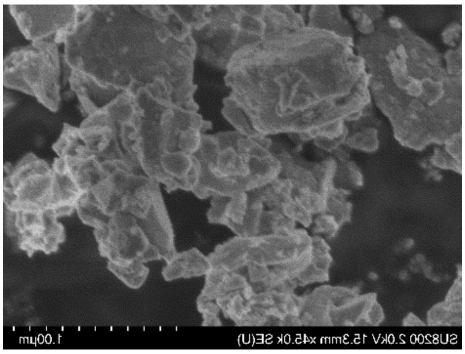

e)

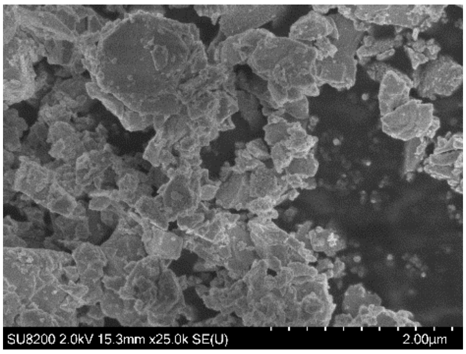

f)

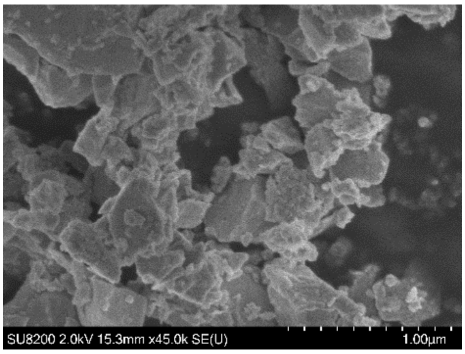

g)

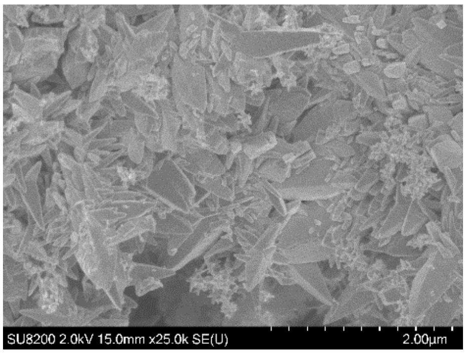

h)

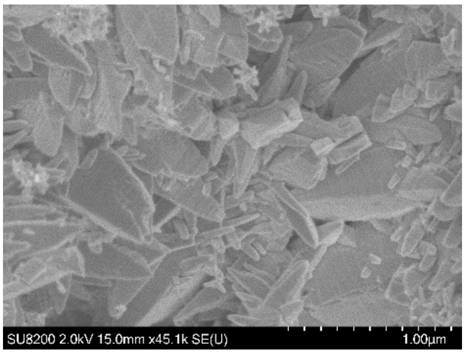

Figure S4: Scanning electron micrographs of BGLC587 (a, b), BGLC82 (c, d), BGLC37 (e, f) and IrO_2 (g, h).

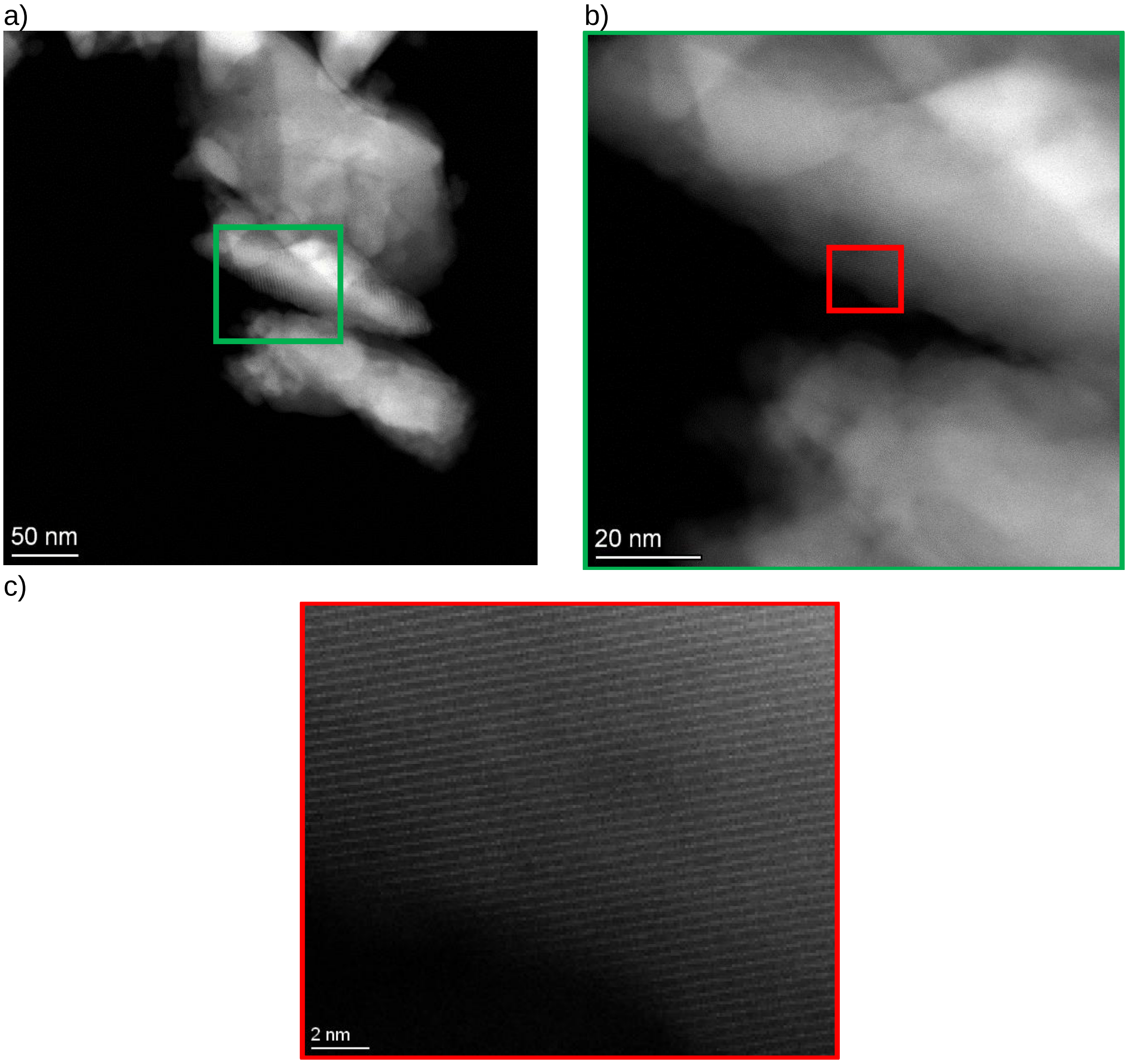

Figure S5: STEM HAADF images of increasing magnifications for BGLC587, showing a crystalline surface (c) in the region emphasised on (a) and (b).

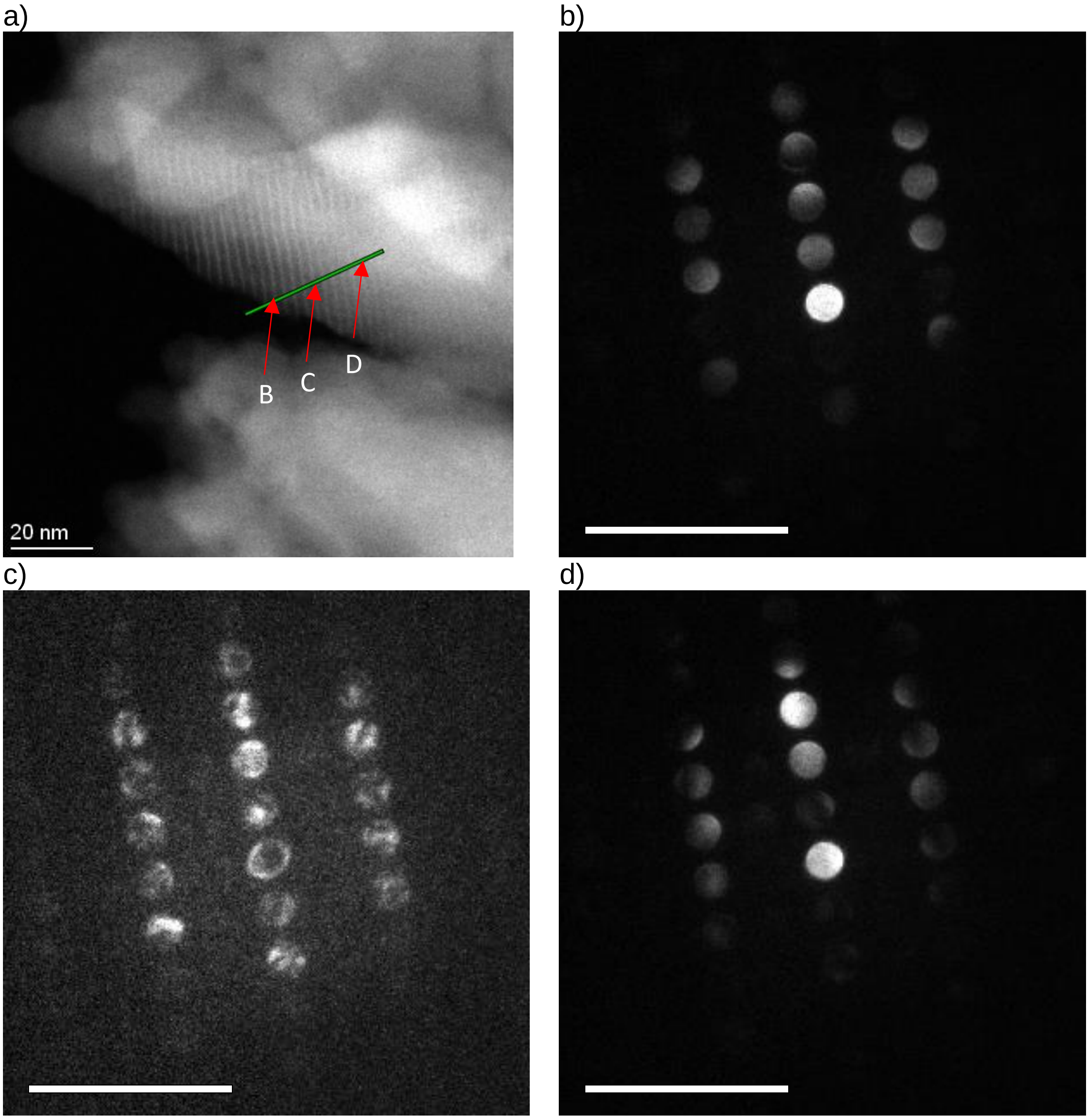

Figure S6: STEM nanobeam scanning diffraction of pre-operation BGLC587 (a). STEM HAADF image taken at regular imaging conditions showing the position of the nanobeam diffraction line profile diffraction patterns extracted from the regions marked B, C, D, showing single grain crystallinity (b-d).

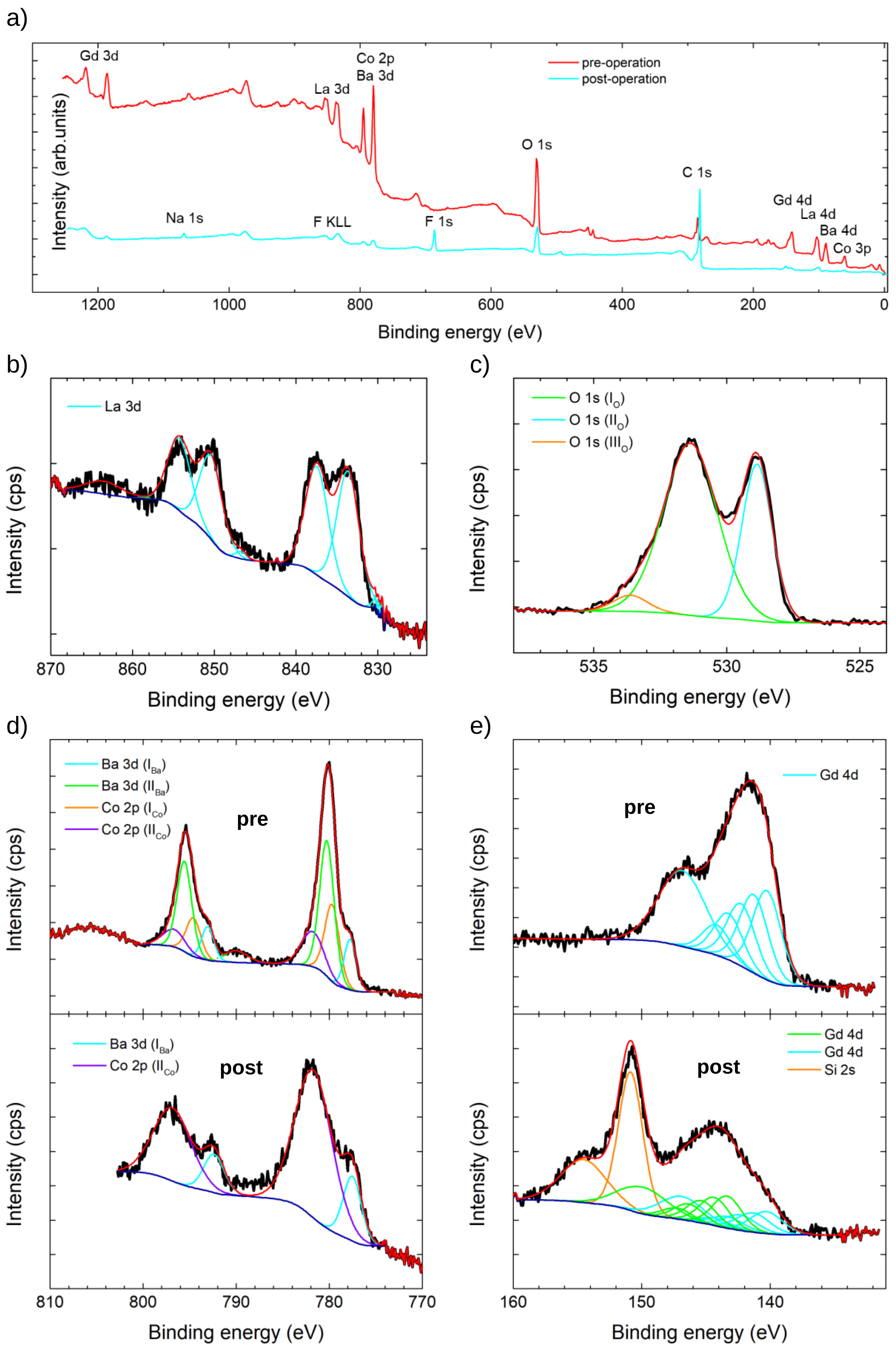

a)
Gd 3d
Co 2p
Ba 3d
La 3d
pre-operation
post-operation
O 1s
C 1s
Gd 4d
La 4d
Ba 4d
Co 3p
Na 1s
F KLL
F 1s
Intensity (arb.units)
Binding energy (eV)
b)
La 3d
Intensity (cps)
Binding energy (eV)
c)
O 1s (I_O)
O 1s (II_O)
O 1s (III_O)
Intensity (cps)
Binding energy (eV)
d)
Ba 3d (I_Ba)
Ba 3d (II_Ba)
Co 2p (I_Co)
Co 2p (II_Co)
pre
Ba 3d (I_Ba)
Co 2p (II_Co)
post
Intensity (cps)
Binding energy (eV)
e)
Gd 4d
pre
Gd 4d
Gd 4d
Si 2s
post
Intensity (cps)
Binding energy (eV)

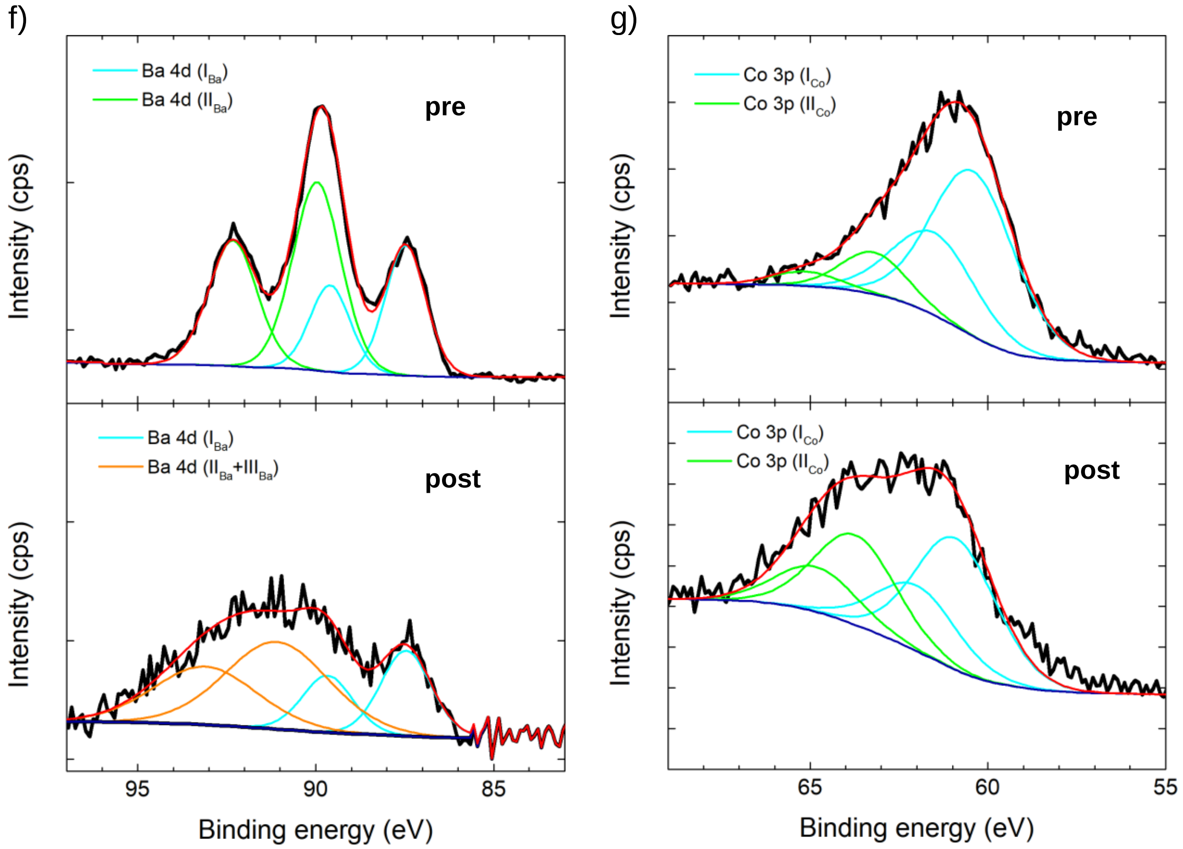

Figure S7: XPS spectra of BGLC587: survey (a), La 3d (b) O 1s (c) Ba 3d/Co 2p (d), Gd 4d (e), Ba 4d (f) and Co 3p (g).

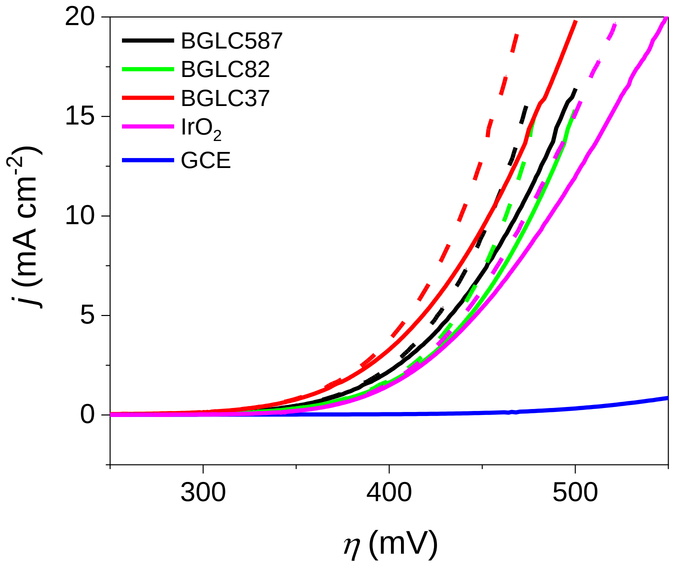

Figure S8: LSV curves at a scanning rate of 10 mV s^{-1} in 1 M NaOH with the *iR*-corrected curves given in dashed lines.

a)

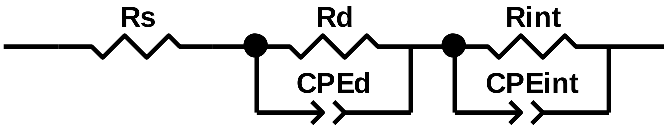

b)

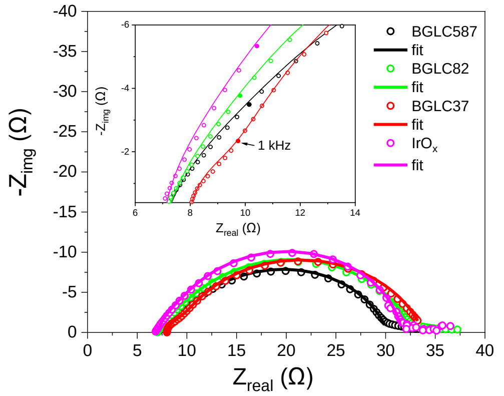

c)

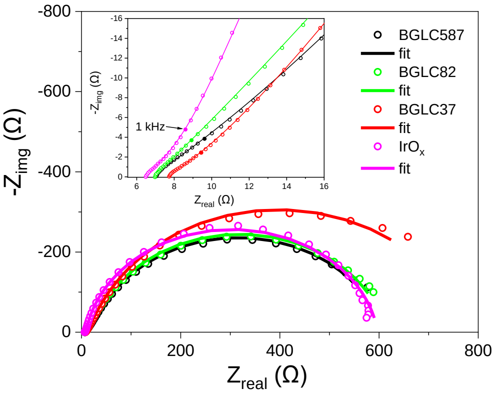

d)

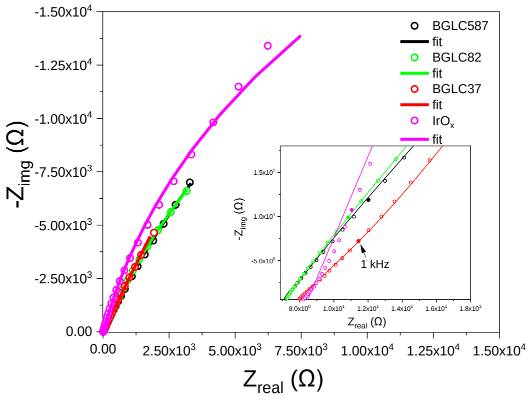

Figure S9: Equivalent circuit used to fit the raw EIS data. All fittings were done in Zview (Scribner) and all χ^2 values were of the order of 10^{-4}. R_s accounts for the solution resistance. R_d and CPE_d account for the resistance and in parallel constant phase element for the diffusion/migration of ionic species in porous electrodes, respectively. This is a simplified approach and is proposed instead of the transmission line model. R_{int} and CPE_{int} account for the interfacial charge transfer resistance and in parallel constant phase element, respectively (a). Nyquist plots of BGLC587, BGLC82, BGLC37 and IrO_2 at potential for 10 mA cm^{-2} (b), onset potential (c) and non-faradaic region (d).

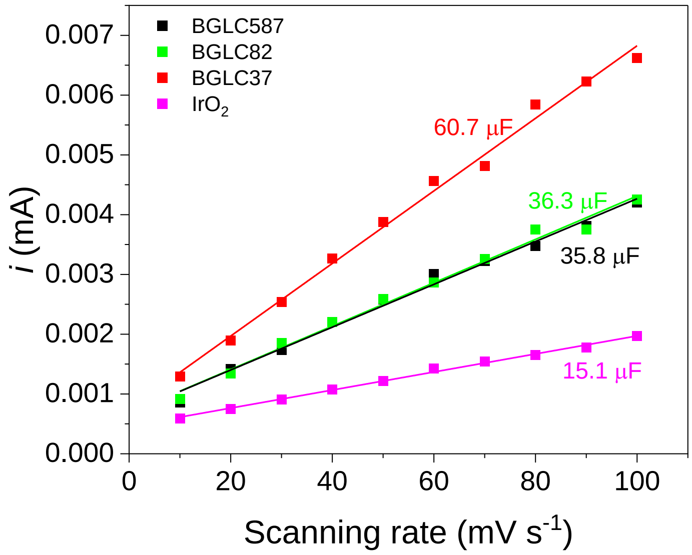

Figure S10: Dependence of the capacitive currents in the CV measurements in a non-faradaic current region from the scan rate for BGLC587, BGLC82, BGLC37 and IrO_2.

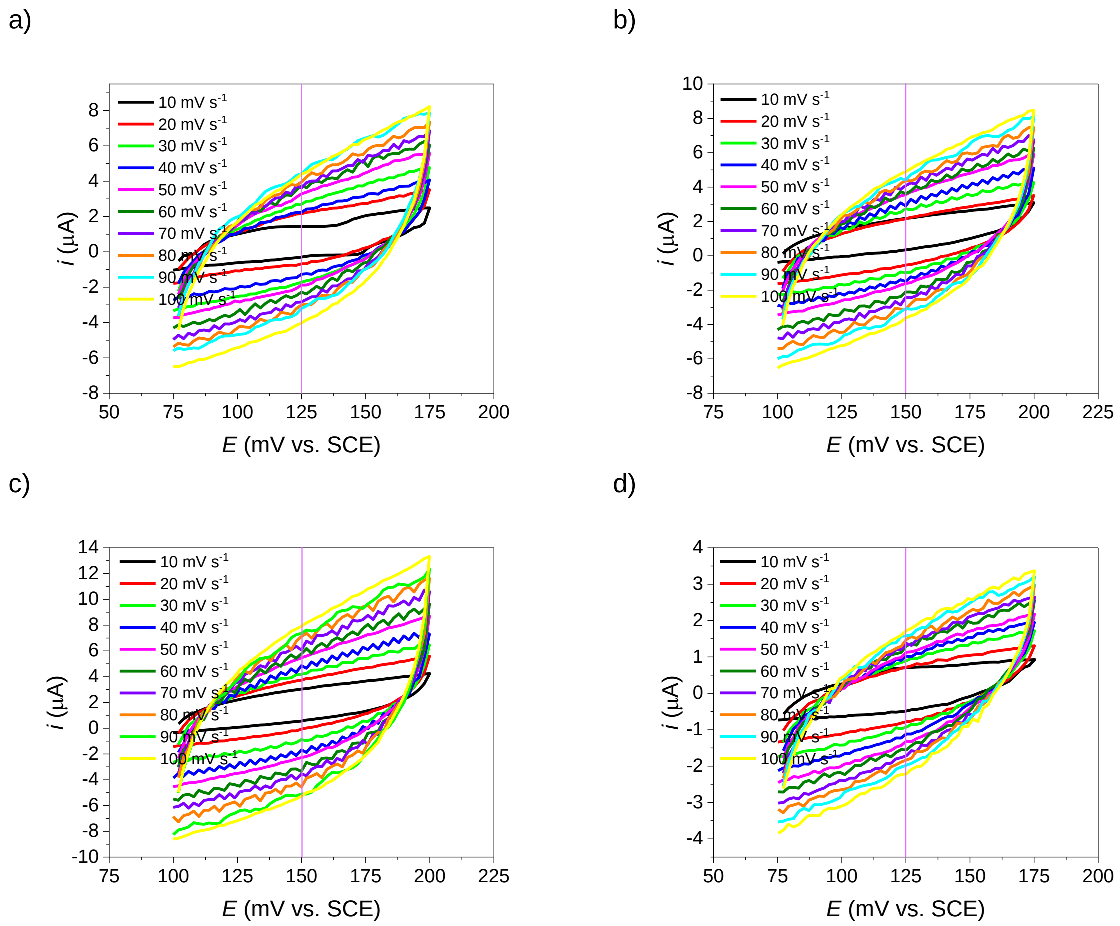

Figure S11: CV measurements in a non-faradaic current region at scan rates from 10 to 100 mV s^{-1} with a 10 mV increment of BGLC587 (a), BGLC 82 (b), BGLC37 (c) and IrO_2 (d) in 1 M NaOH solution.

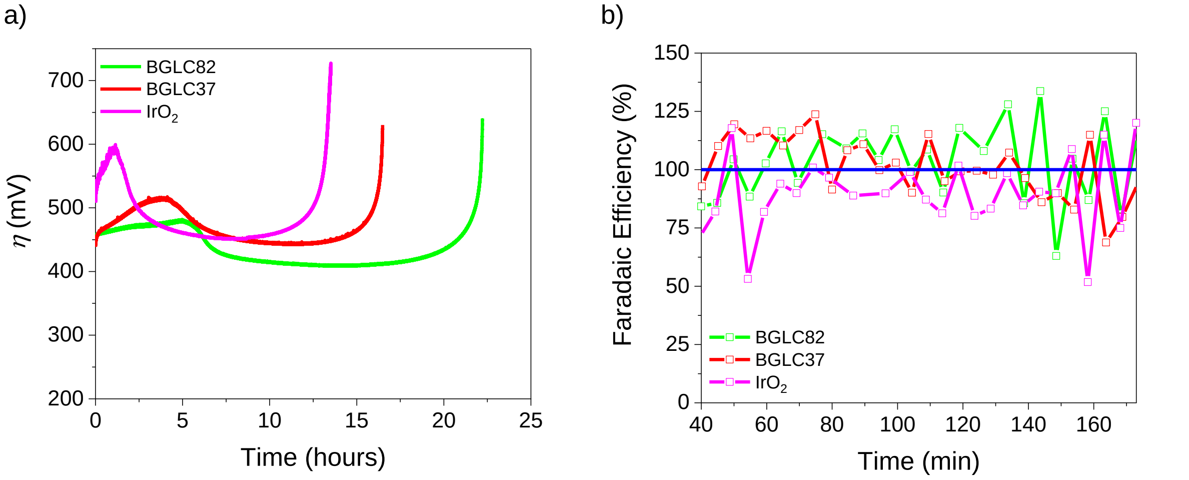

Figure S12: Galvanostatic stability experiments for BGLC82, BGLC37 and IrO_2 at 10 mA cm^{-2} at 1000 rpm and 0.280 mg of catalyst. Hg/HgO (1 M NaOH) was used as the reference electrode (a). Faradaic efficiency of BGLC82, BGLC37 and IrO_2 on carbon paper loaded with approx. 5 mg cm^{-2}. A few minutes in the beginning are needed in order to stabilise the measurement, while the irregular gas release and accumulation in the headspace of the measuring cell lead to fluctuations in the FE (b).

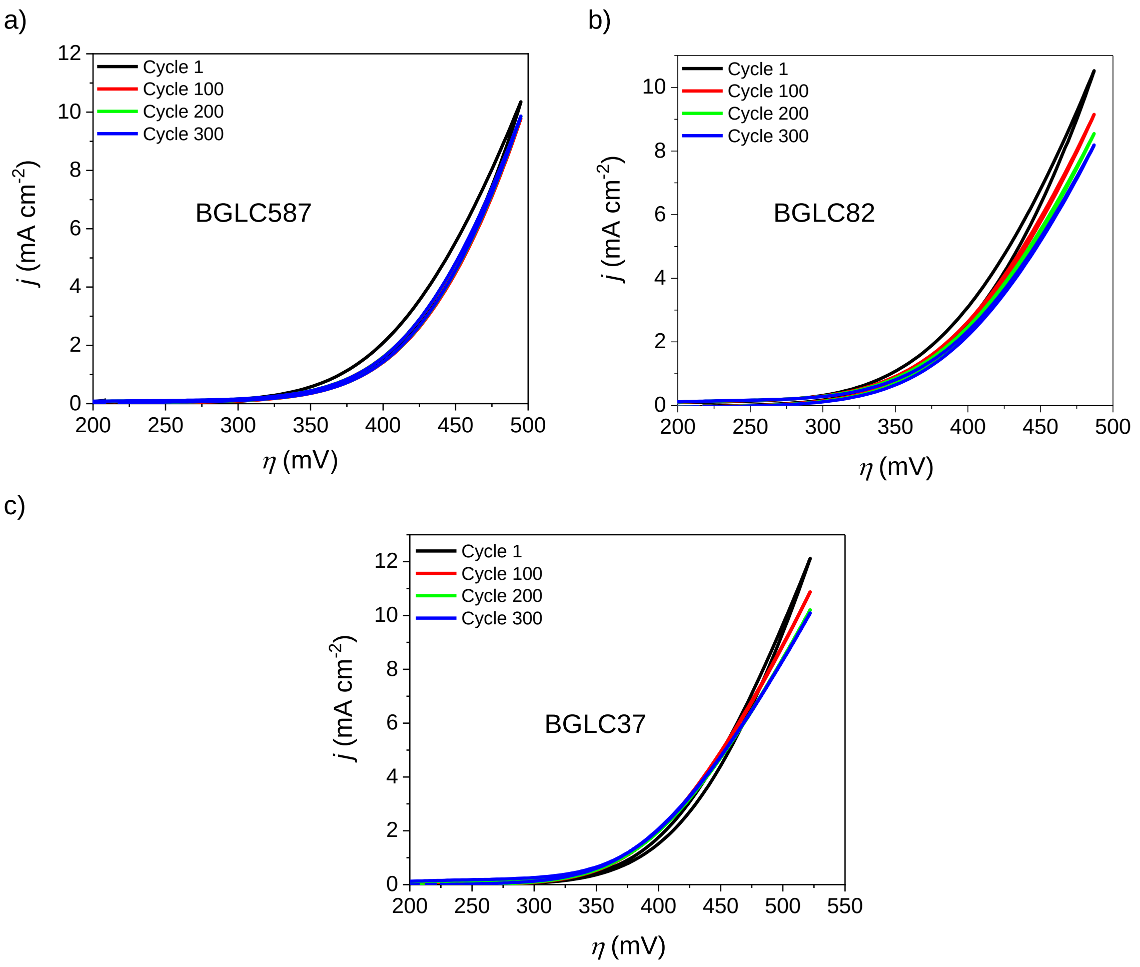

Figure S13: CV scans of BGLC587 (a), BGLC82 (b) and BGLC37 (c) loaded on carbon paper (~2 mg $cm^{-2}{}_{nominal\ area}$). Scan rate 10 mV s^{-1} in 1 M NaOH.

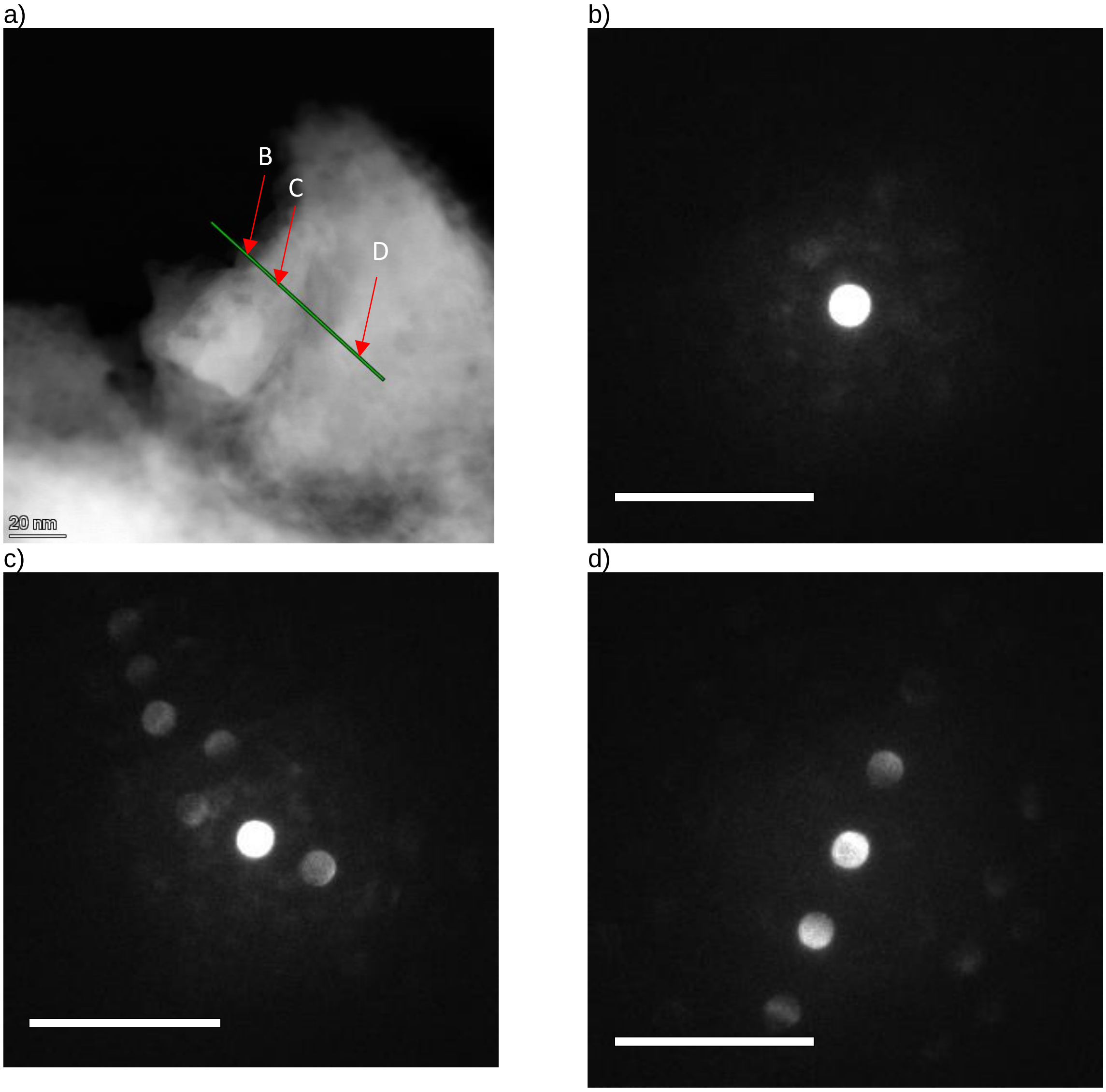

Figure S14: STEM nanobeam scanning diffraction of Post operation BGLC587 (a). STEM HAADF image taken at regular imaging conditions showing the position of the nanobeam diffraction line profile diffraction patterns extracted from the regions marked B, C, D on (a), showing a 5 nm thick amorphous surface layer (b) and crystalline core (c, d).

a)

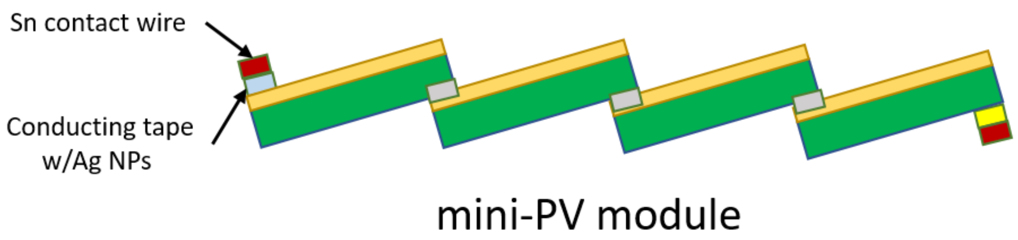

b)

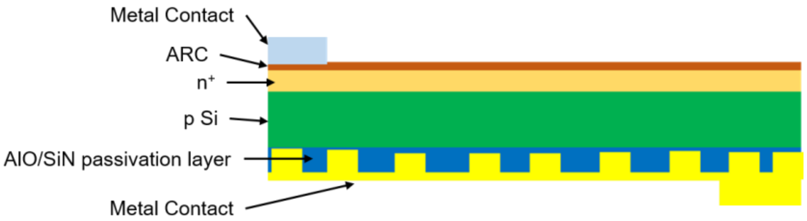

Figure S15: Schematic presentation of the mini-PV module configuration. The solar cells were laser cut in 1 x 1.2 cm and an overlap of 0.2 x 1 cm was utilised in order to fasten them together by Ag NPs filled conducting tape. The same way Sn contact wires were also attached (a). Schematic cross section of the individual Si solar cells used in the mini-PV module (b). ARC: Anti Reflection Coating.

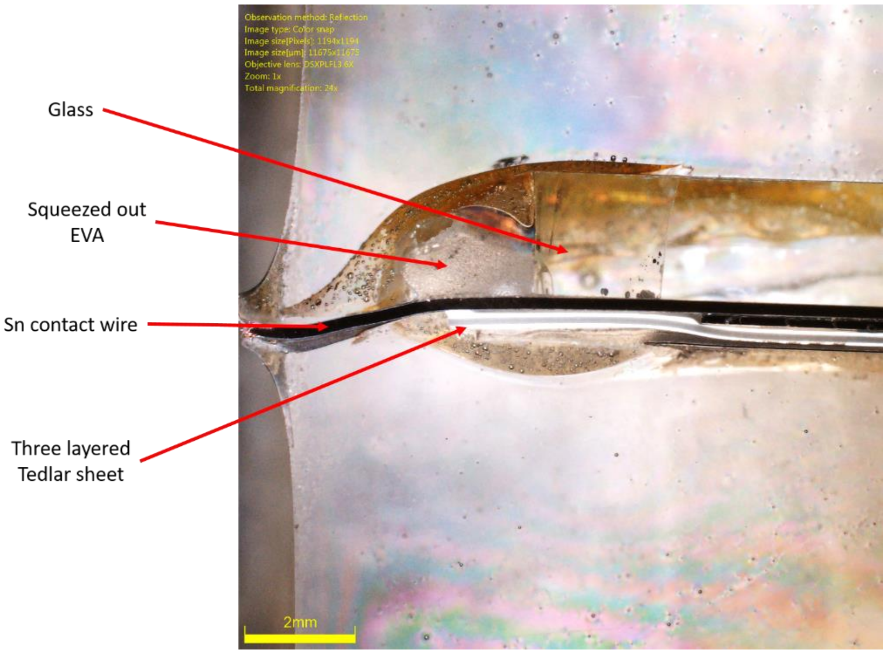

Figure S16: Lower magnification of the ea-PV-PEC device cross section of Fig. 3c in the main document, where the Sn contact wire, Tedlar sheet and glass can be seen.

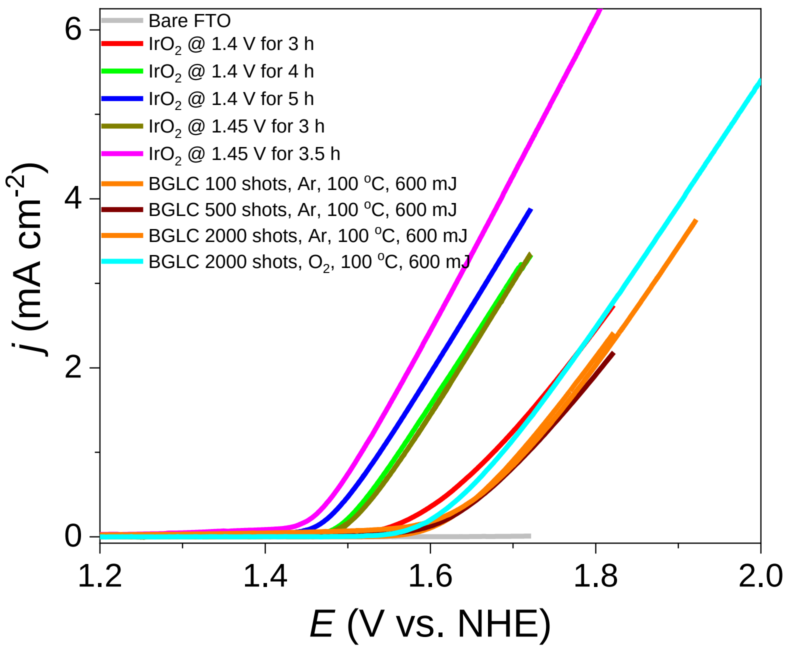

Figure S17: Optimisation of the electrodeposited IrO_2 and PLD-deposited BGLC587 on FTO. It can be seen that IrO_2 deposited at 1.45 V vs. SCE from the colloidal solution of IrO_2 nanoparticles for 3.5 h gave the best performing catalyst layer. For BGLC587, the best performance was obtained for PLD deposition parameters of 2000 shots under pure O_2 atmosphere, at substrate temperature of 100 °C and a laser energy of 600 mJ.

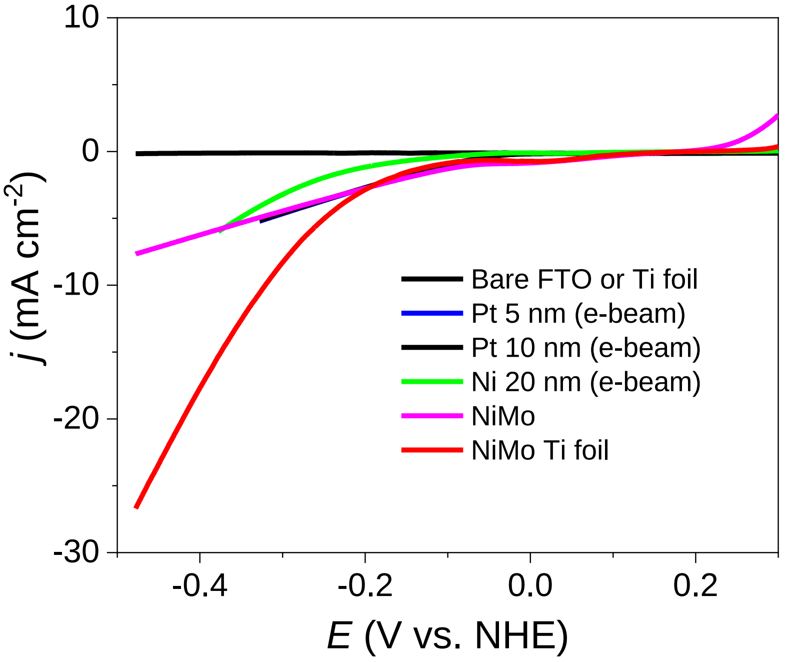

Figure S18: Current-voltage curves of different catalysts for the HER in 1 M NaOH at 10 mV s^{-1}. Except for the NiMo Ti foil curve (red) the rest of the catalysts were tried on FTO-coated glass. Pt of 5 and 10 nm deposited by ebeam deposition, as well as Ni of 20 nm, were compared against electrodeposited NiMo on FTO and Ti foil. The optimised electrodeposition process is described in the experimental in the main document. Briefly, a 2-electrode set up was used with Ni foam serving as a sacrificial anode and Ti foil or FTO as the substrate (cathode) in a Ni, Mo ion plating solution in pH of approx. 10.5-11.0.

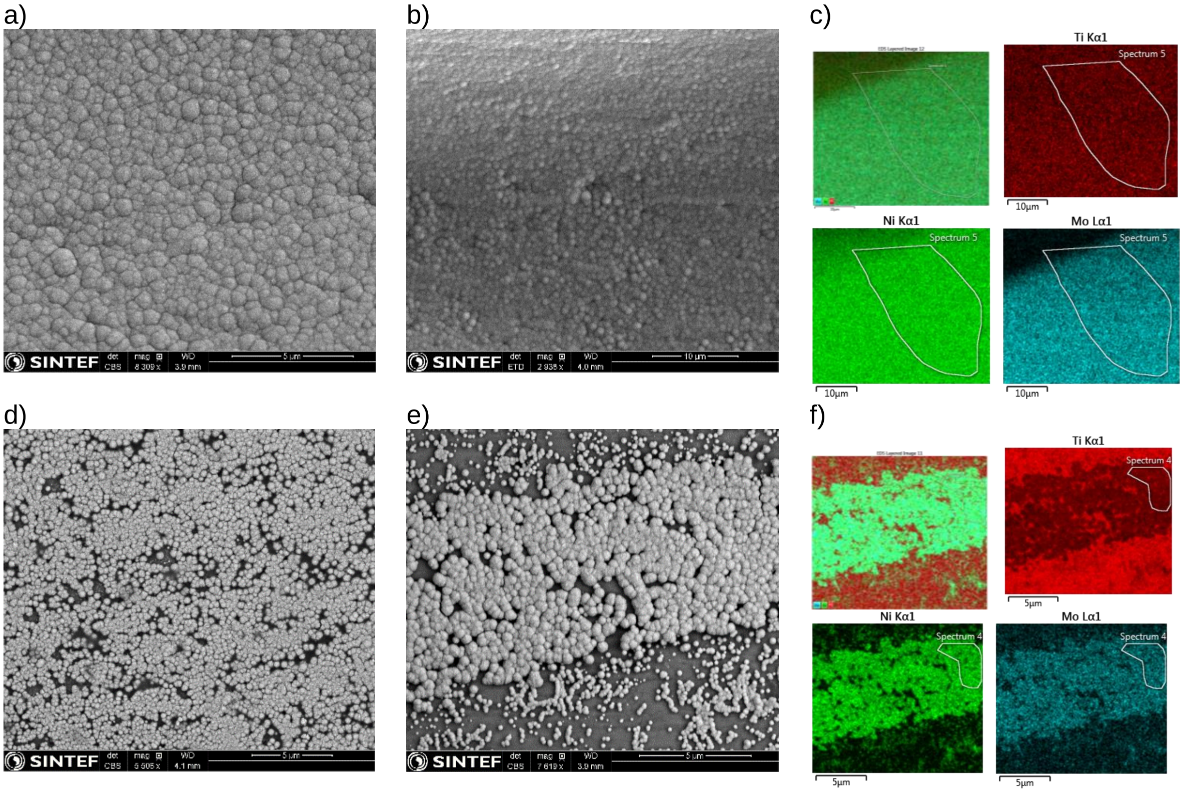

Figure S19: SEM observation of fresh NiMo deposits on Ti foil (a, b) and its EDS analysis (c). The used NiMo electrode after 71 h of laboratory and 8 h outdoors operations (d, e) and its EDS analysis (f). Significant loss in deposits can be seen.

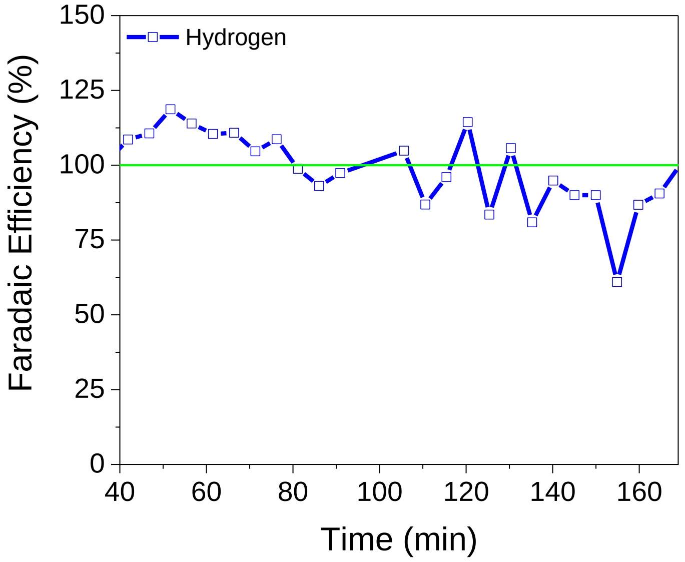

Figure S20: Faradaic efficiency of the NiMo on Ti foil. A few minutes in the beginning are needed in order to stabilise the measurement. The fluctuations are due to irregular gas accumulation in the headspace of the electrolytic cell.

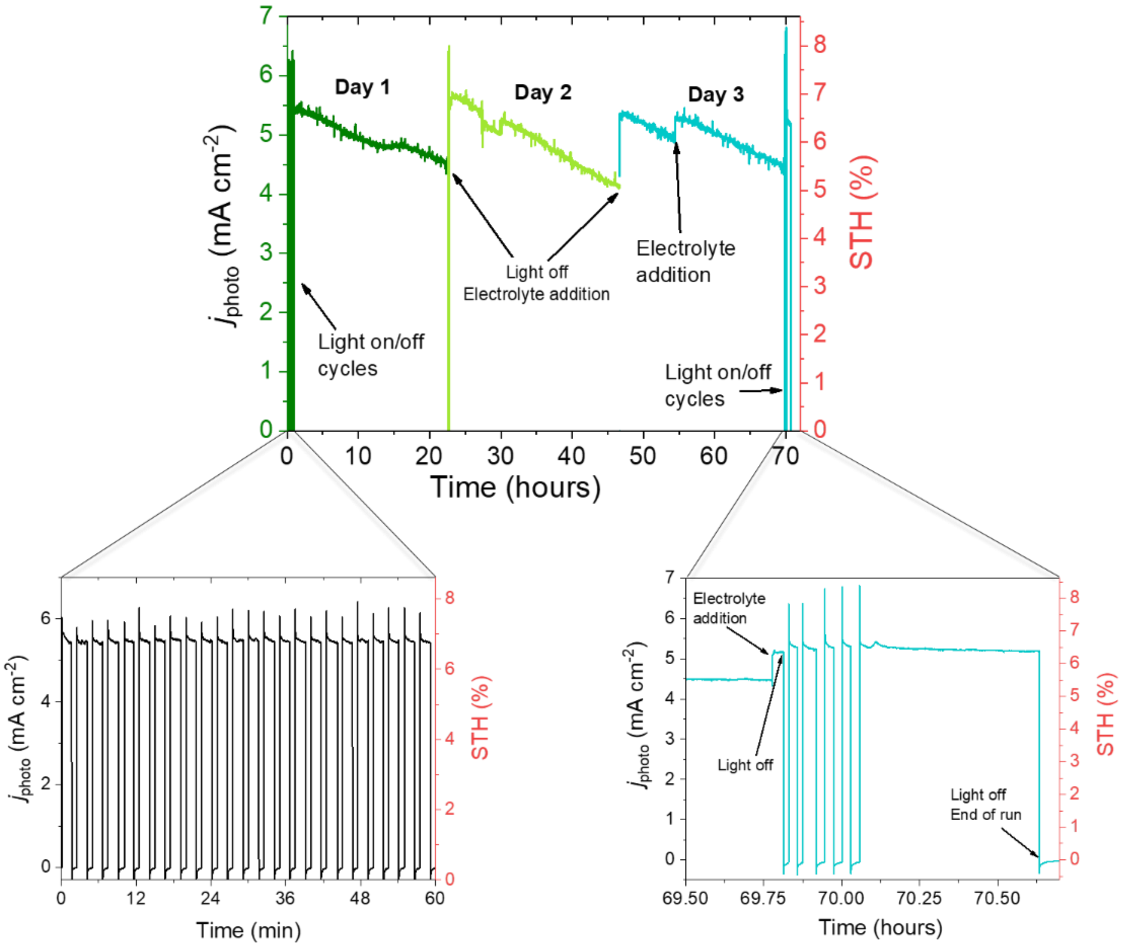

Figure 21: A duplicate assembly where light on/off cycles and electrolyte evaporation are highlighted.

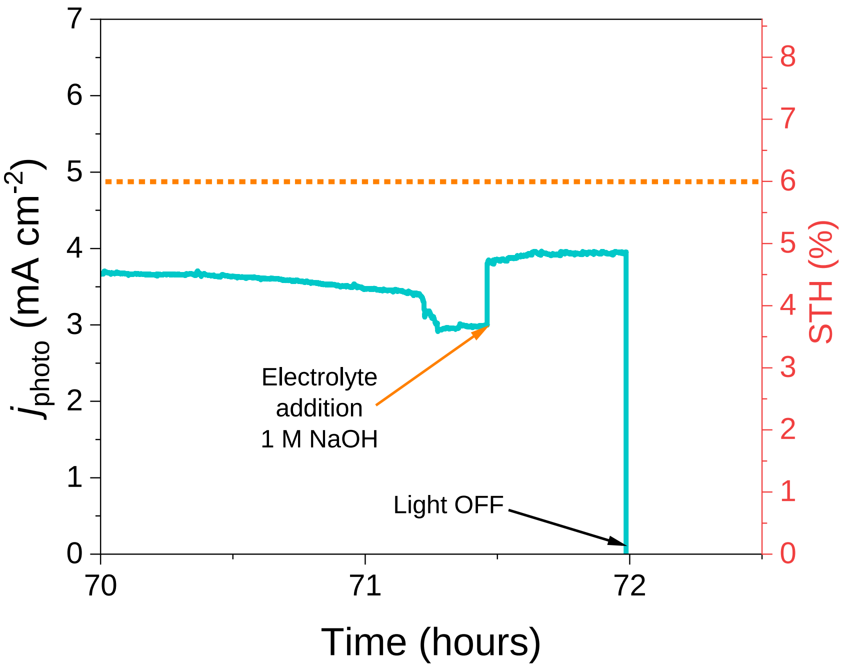

Figure S22: Increased magnification of Fig. 4c of the main document, where the irreversible loss in the water splitting activity of the ea-PV-PEC device can be seen a few minutes after the 71 h mark. Electrolyte addition did not retrieve the STH of 6.64%, but it is rather reduced to approx. 5%.

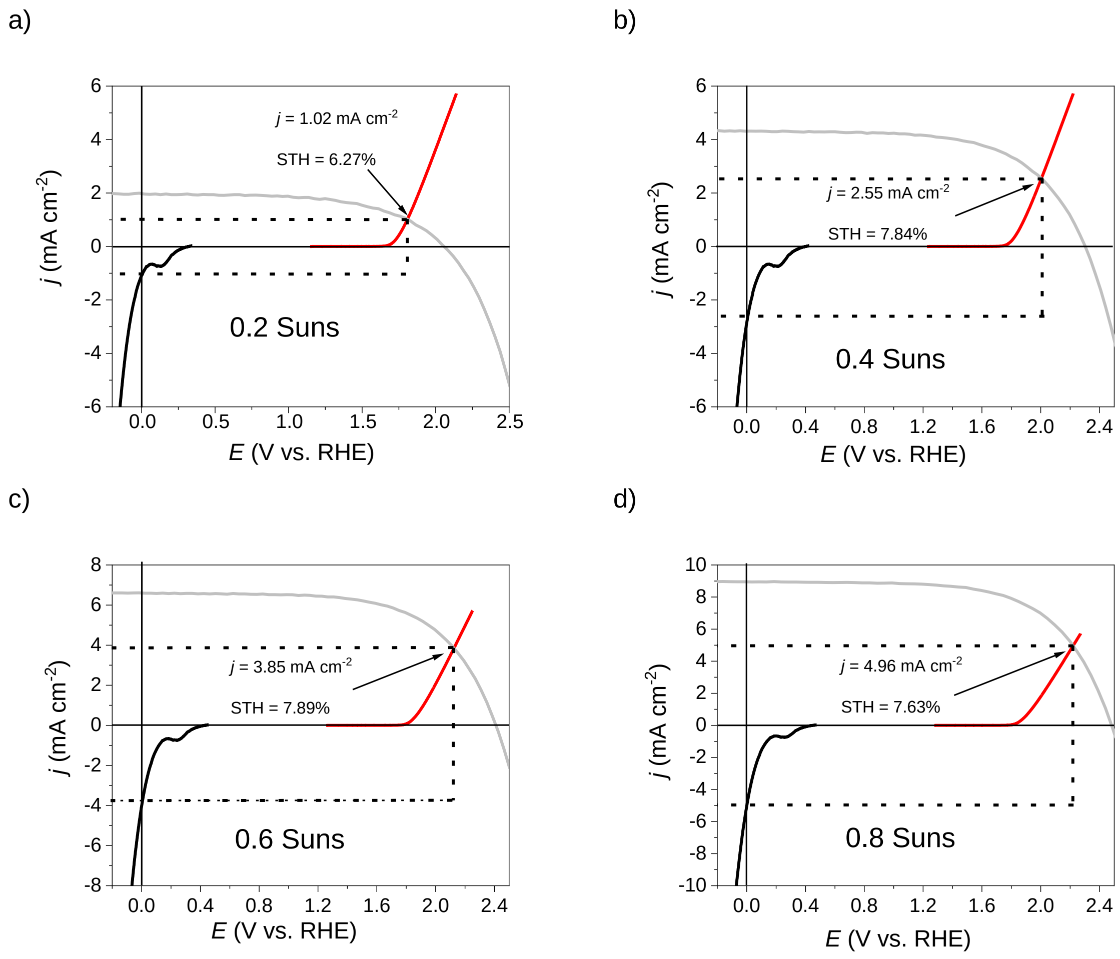

Figure S23: Current-voltage curve of the Si-MM under varying solar simulated illuminations (0.2, 0.4, 0.6 and 0.8 Suns) and the current-potential curves of the BGLC587 anode on FTO, NiMo cathode on Ti foil, and the expected photocurrent density and STH efficiencies.

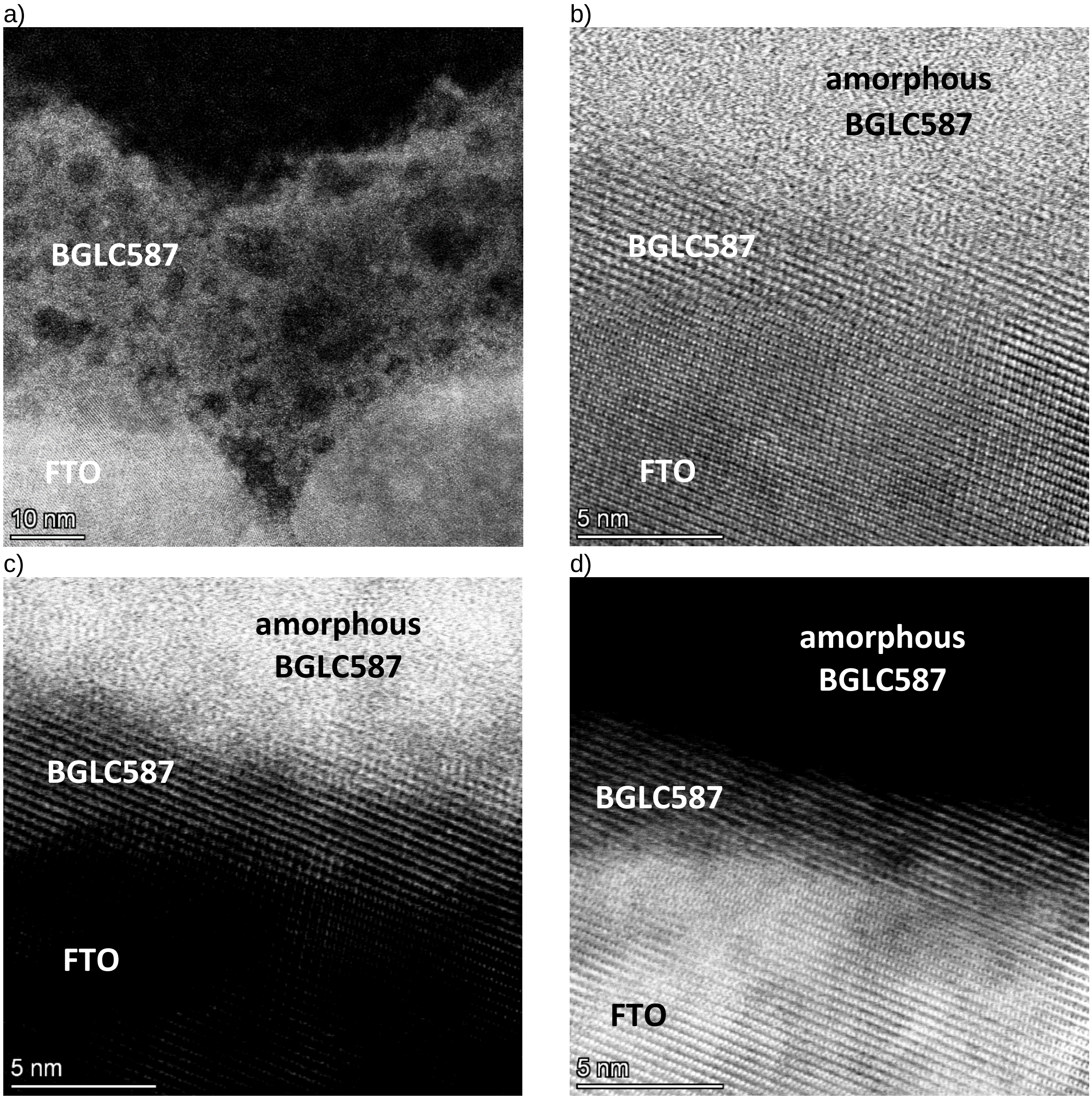

Figure S24: HAADF HRSTEM bright field images of BGLC587 after 71 h of laboratory operation and 8 h outdoors operation (a). Images b, c and d are obtained under different contrast to highlight the amorphisation of BGLC587. A crystalline layer of only ~2 nm of BGLC can still be seen.

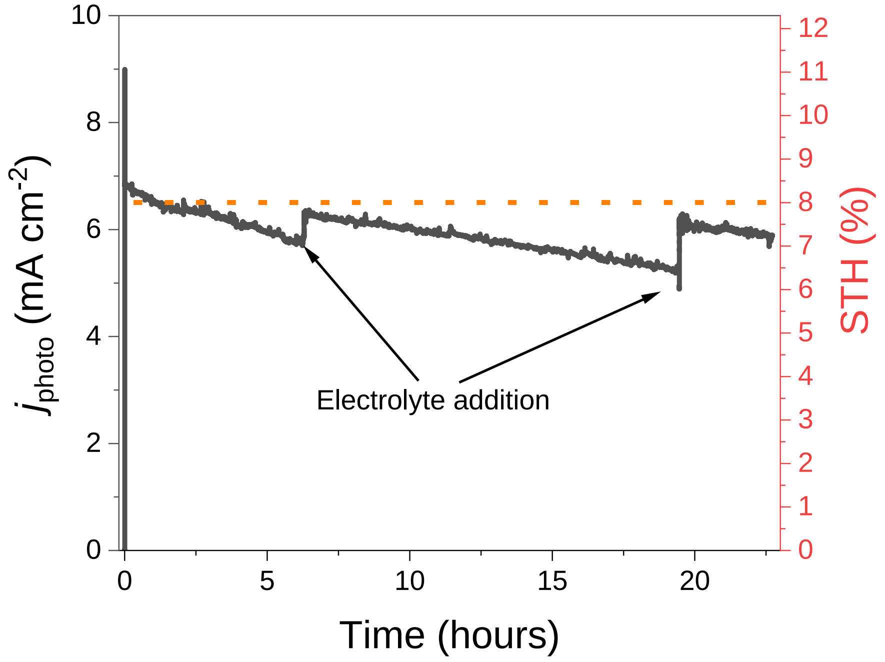

Figure S25: Indoors bias free photoelectrolysis of water under 1 Sun simulated illumination in 1 M NaOH. The PV-PEC device is assembled the same way as described in Fig. 3 in the main document, with the difference that instead of BGLC deposition by PLD, IrO_2 is electrodeposited on the FTO. The conditions were 1.45 V vs. SCE for 3.5 h in the colloidal solution of IrO_2. In this case and after each electrolyte compensation the photocurrent density is not fully retrieved.

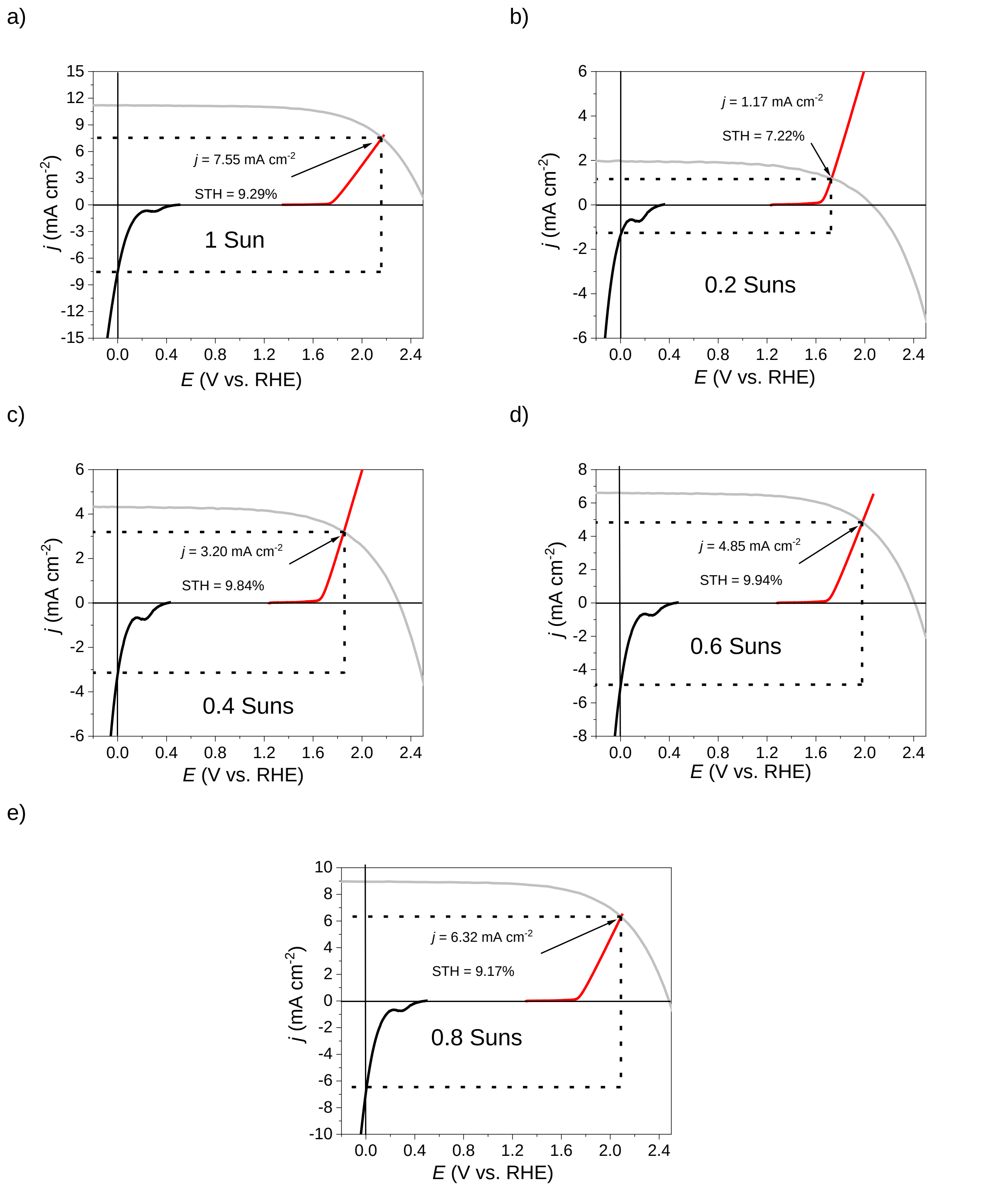

Figure S26: Current-voltage curve of the Si-MM under varying solar simulated illuminations (0.2, 0.4, 0.6 and 0.8 Suns) and the current-potential curves of the IrO_2 anode on FTO, NiMo cathode on Ti foil, and the expected photocurrent density and STH efficiencies.

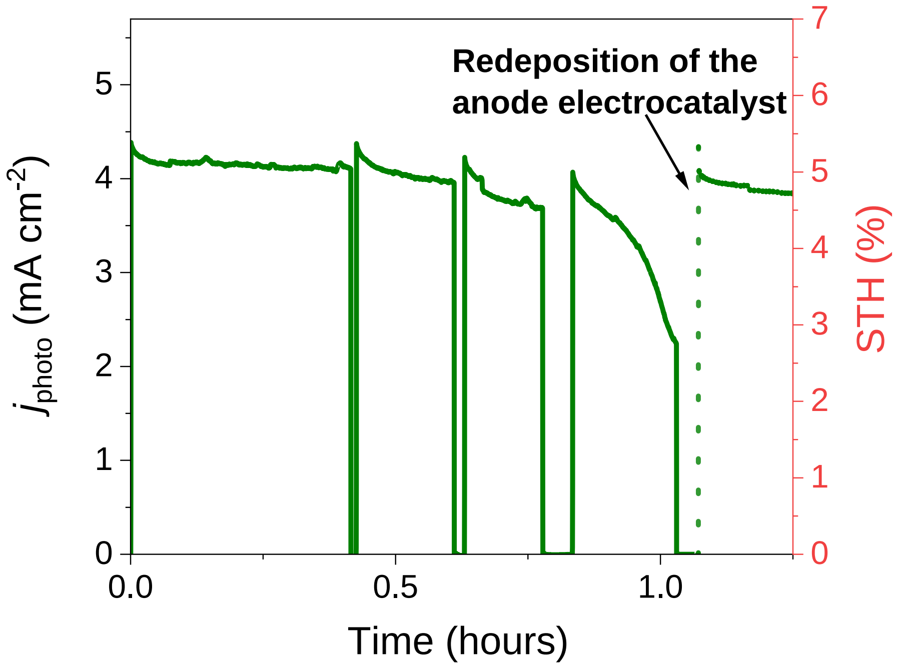

Figure S27: Indoors bias free photoelectrolysis of water under 1 Sun simulated illumination in 1 M NaOH. The anode in this case was electrodeposited IrO_2 and close to the 1 h mark, the performance of the monolithic device was significantly reduced. The anode catalyst was electrodeposited again and the performance was fully retrieved, clearly suggesting the dissolution of IrO_2 in the first place [1, 2]. This device was operated with a non-optimised NiMo-based cathode catalyst.

a)

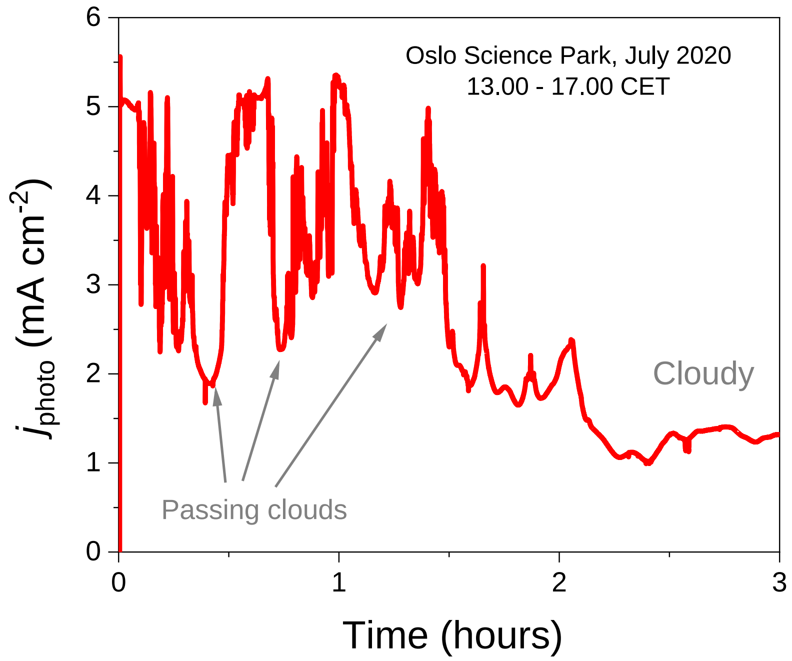

b)

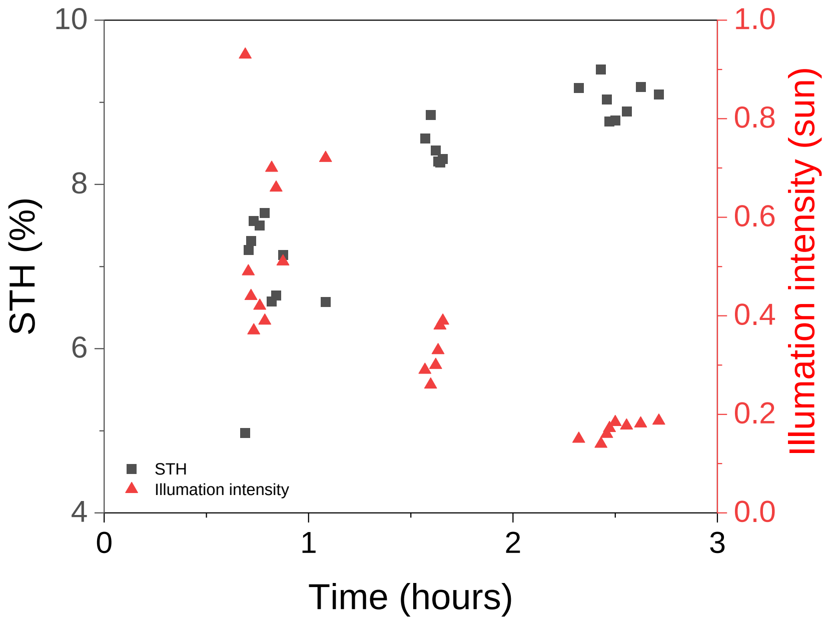

Figure S28: Outdoors operation in 1 M NaOH under varying light intensity conditions due to passing clouds for the IrO_2 driven PV-PEC device, (a). Outdoors STH efficiency under varying light intensity conditions, corresponding directly to a), (b).

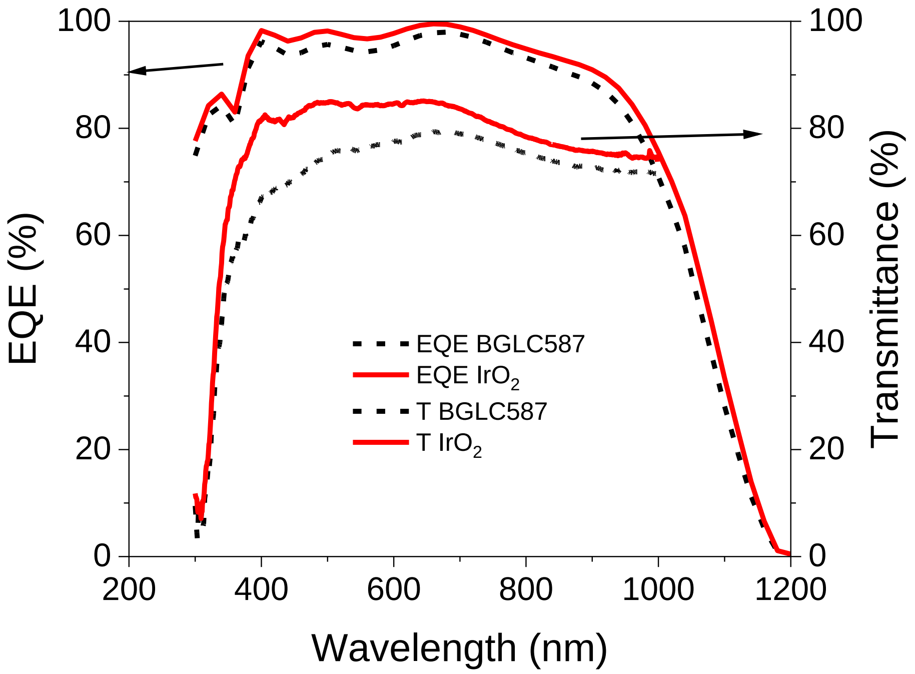

Figure S29: EQE and transmittance spectrum of IrO_2 coated FTO glass by electrodeposition at 1.45 V vs. SCE for 3.5 h. The EQE and transmittance spectrum of BGLC587 is also given for comparison purposes in dashed lines. The BGLC587 curves are the same with those in Fig. 4b in the main document.

List of Tables

Table S1: Sum spectrum EDS compositions for the pre- and post-operation (48 h) BGLC587 catalyst.

**Element**	**Atomic fraction (%)**	**Atomic error (%)**	**TEM region**
Pre-operation			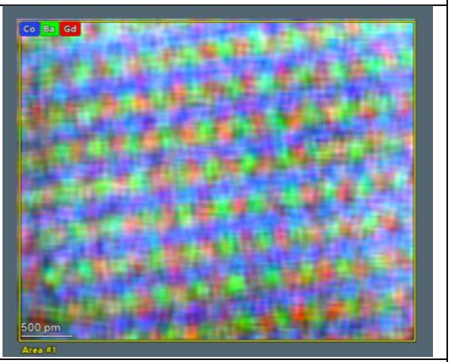
Co	52.89	5.46	
Ba	18.94	2.56	
La	11.6	1.57	
Gd	16.56	2.25	
Post-operation			
Co	57.96	6.15	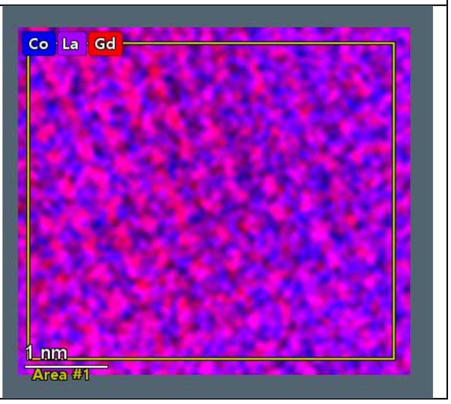
Ba	4.53	0.62	
La	21.65	2.98	
Gd	15.86	2.19	

Table S2: XPS compositions for pre-operation BGLC587 catalyst.

XPS region	Atomic fraction (%)
Co 3p	39
Ba 4d	13
La 3d	26
Gd 4d	22

Table S3: Relative XPS compositions based on Co 3p and Ba 4d for the pre- and post-operation BGLC587 catalyst, compared to results from Table S1.

	Atomic fraction (%)			
	XPS		EELS (from Table S1)	
	Ba	Co	Ba	Co
Pre-operation	25	75	26	74
Post-operation	9	91	7	93

Table S4: Electrochemical parameters as extracted by the EIS measurements at potentials for 10 mA cm^{-2}. The parameters are coupled to the fitted data of Fig. 9b.

**Catalyst**	**R_s (Ω)**	**R_d (Ω)**	**$Y(0)_d$ (F)**	**n_d**	**R_{int} (Ω)**	**$Y(0)_{int}$ (F)**	**n_{int}**
BGLC587	7.1	19.7	$2.48\ 10^{-4}$	0.83	3.2	$3.59\ 10^{-4}$	0.74
BGLC82	7.2	23.4	$1.94\ 10^{-4}$	0.83	1.8	$2.94\ 10^{-4}$	0.81
BGLC37	8.0	24.0	$3.95\ 10^{-4}$	0.82	1.7	$3.19\ 10^{-4}$	0.82
IrO_2	7.1	41.0	$0.47\ 10^{-4}$	0.87	0.9	$0.36\ 10^{-4}$	1.0

Table S5: Electrochemical parameters as extracted by the EIS measurements at the onset potentials for 0.3 mA cm^{-2}. The parameters are coupled to the fitted data of Fig. 9c.

**Catalyst**	**R_s (Ω)**	**R_d (Ω)**	**$Y(0)_d$ (F)**	**n_d**	**R_{int} (Ω)**	**$Y(0)_{int}$ (F)**	**n_{int}**
BGLC587	6.6	37.3	0.00287	0.52	563	$3.3\ 10^{-4}$	0.87
BGLC82	6.8	32.5	0.0022	0.58	576	$2.6\ 10^{-4}$	0.88
BGLC37	7.4	52.9	0.0069	0.47	717	$4.8\ 10^{-4}$	0.828
IrO_2	6.4	3.9	0.0022	0.57	587	$0.9\ 10^{-4}$	0.91

The exponential value of the CPE_d parameter is approx. 0.5, which clearly indicates this constant phase element has a Warburg behaviour, consistent with a purely diffusive process. Therefore, the C_{dl} parameter in the onset potentials was found by the interfacial CPE element alone. In fact, even if the effective capacitance is extracted, it is so large that it does not contribute to the total effective capacitance of the double layer.

Table S6: Electrochemical parameters as extracted by the EIS measurements at non-faradaic current regions. The parameters are coupled to the fitted data of Fig. 9d.

**Catalyst**	**R_s (Ω)**	**R_d (Ω)**	**$Y(0)_d$ (F)**	**n_d**	**R_{int} (Ω)**	**$Y(0)_{int}$ (F)**	**n_{int}**
BGLC587	6.9	8.8	$6.7\ 10^{-4}$	0.74	79072	$1.0\ 10^{-4}$	0.78
BGLC82	7.0	6.9	$8.7\ 10^{-4}$	0.73	59741	$1.0\ 10^{-4}$	0.79
BGLC37	7.7	10	$7.0\ 10^{-4}$	0.71	120300	$1.7\ 10^{-4}$	0.77
IrO_2	8.4	-	-	-	58002	$0.6\ 10^{-4}$	0.87

Using this approach as $R_{ct} = R_d + R_{int}$ and after the CPEs are transformed into capacitances. The effective capacitance was determined by $C_i = \left[Y_i R_i^{(1-n_i)}\right]^{1/n_i}$. Thereafter, $C_{dl} = \frac{1}{\frac{1}{C_d}+\frac{1}{C_{int}}}$. It should be noted that a simple Randles circuit was used in the case of IrO_x, where it provided much better fitting results.

Table S7: Comparison between capacitance values as obtained by cyclic voltammetry at the potential range 1.0-1.1 V vs. NHE, and capacitance values as extracted by EIS at two potential regions. $R_{ct}C_{dl}$ parameter

**Catalyst**	**Capacitance from CV scanning (μF)**	**Capacitance from EIS at the CV scanning potentials (capacitive region) (μF)**	**Capacitance from EIS at onsets (μF)**	**Capacitance at 10 mA cm^{-2} (OER region) (μF)**	**Relative decrease from non-faradaic to OER regions**
BGLC587	35.8	70	148	24	66%
BGLC82	36.3	75	124	28	63%
BGLC37	60.7	80	350	42	47%
IrO_2	15.1	55	18	17	69%

Table S8: Performances of previously reported state-of-the-art perovskite catalysts supported on glassy carbon disc electrode for the OER in alkaline media.

**Catalyst**	**Overpotential (mV)**	**Tafel (mV dec^{-1})**	**Stability**	**Amount**	**Ref**
$SrFeO_3$	400	65-70	24,<br>some degradation	-	[3]
$Ba_{0.5}Sr_{0.5}Co_{0.8}Fe_{0.2}O_{3-\delta}$	500	129	N/A	0.64 mg cm^{-2}	[4]
$(Pr_{0.5}Ba_{0.5})CoO_{3-\delta}$	350 (@ 5 mA cm^{-2})	60	2 h,<br>some degradation	0.25 mg cm^{-2}	[5]
$PrBa_{0.5}Sr_{0.5}Co_{1.5}Fe_{0.5}O_{5+\delta}$	358	52	12 h,<br>some degradation	0.20 mg cm^{-2}	[1]
$(PrBa_{0.85}Ca_{0.15})_{0.5}(MnFe)_{0.5}O_{3-\delta}$	410	86	N/A	0,28 mg cm^{-2}	[6]
RP-$LaSr_3Co_{1.5}Fe_{1.5}O_{10-\delta}$ /<br>$La_{0.25}Sr_{0.75}Co_{0.5}Fe_{0.5}O_{3-\delta}$	324	58	2.7 h,<br>5% degradation	0.25 mg cm^{-2}	[7]
$Cs_{0.4}La_{0.6}Mn_{0.25}Co_{0.75}O_3$	400	101	12 h,<br>2% degradation	0.45 mg cm^{-2}	[8]
$BaGdCo_{1.8}Fe_{0.2}O_3$	477	60	48 h,<br>3% degradation	4.00 mg cm^{-2}	[9]
$CaCu_3Fe_4O_{12}$	380	51	N/A	0.25 mg cm^{-2}	[10]
RP-$La_{0.5}Sr_{1.5}Ni_{1-x}Fe_xO_{4\pm\delta}$	360	44	N/A	15.3 μg_{ox} cm^{-2}	[11]
$SrCo_{0.5}Si_{0.5}O_{3-\delta}$	417	66	N/A	0.255 mg cm^{-2}	[12]
$Ba_{0.5}Gd_{0.8}La_{0.7}Co_2O_{6-\delta}$	470	78	48 h<br>no degradation from *j-t*<br>some degradation from STEM	0.28 mg cm^{-2}	This work

Table S9: EDS quantification of the NiMo layers from Fig. S19 obtained from the selected areas in each sample

Sample	Ni	Mo	Ti	Ni:Mo
Fresh NiMo layer (b from Fig. S14)	85.26	10.07	4.67	8.5
Post operation NiMo layer (e from Fig. S14	41.58	3.93	54.33	10.6

Table S10: Configurations and performances of previously reported bias-free photoelectrochemical water electrolysis cells.

Device configuration (Anode‖Cathode)	Monolithic (M), Wired (W), Monolithic but Wired (MW)	All earth-abundant catalysts, Device size	Electrolyte , Illumination conditions	STH efficiency (%)	Stability, Notes	Work, Date
BGLC587/FTO‖ Si mini module/Ti-foil/NiMo, PV "buried" in tandem	M	Yes,<br>Mini module: 4 cm^2<br>Electrodes: 4 cm^2	1 M NaOH,<br>1 Sun	6.64	71 h,<br>No degradation	This work
Pt‖GaInP(p)/GaAS(n)/Pt	MW	No,<br>-	3 M H_2SO_4, 0.01 M Triton X,<br>11 Suns	12.4	20 h,<br>20% drop in current	[13],<br>1998
RuO_2‖$Al_{0.15}Ga_{0.85}As$(pn)/Si(pn)/Pt	W	No,<br>Illuminated: 0.22 cm^2<br>Electrolysis: 10 cm^2	1 M $HClO_4$<br>135 mW cm^{-2}	18.3	14 h	[14],<br>2000
CoPi/$BiVO_4$/a-Si:H(pin)/nc-Si:H(pin)‖Pt wire	MW	Partially,<br>0.283 cm^2	Neutral pH<br>1 Sun	5.2	1 h,<br>Less than 5% drop in current	[15],<br>2014
WO_3/FTO/pn-Si‖np-Si/Ti/TiO_2/Pt	M	Partially,<br>2 x 7.04 cm^2	1 M $HClO_4$, Nafion membrane,<br>2 Suns	0.24	20 h,<br>Stable H_2 generation	[16],<br>2015
Pt‖GaInP(pn)/InGaAs(pn)/Ge(pn)/Pt	W	No,<br>Illuminated: 1 cm^2<br>Electrolysis: 4 cm^2	Neutral pH<br>10 Suns	15	1 h,<br>3 solar cells and 2 PEM electrolysers	[17],<br>2013
FTO/a-Si(pin)/ a-Si(pin)/ a-Si(pin)‖Pt	W	Partially,<br>1 cm^2	5 M KOH,<br>1 Sun	6.2	31 days,<br>Stable current density	[18],<br>2006
Pt‖Pt connected to CIGS<br>3 cells wired side by side	M	No,<br>-	3 M H_2SO_4,<br>1 Sun	10	27 h	[19],<br>2013
NiFe‖NiFe connected to 2 halide perovskites (Pb co/containing)	W	Yes,<br>Perovskite: 0.159 cm^2<br>Electrodes: 1 cm^2	1 M NaOH,<br>1 Sun	12.3	2 h,<br>Stable photocurrent	[20],<br>2014
$NiFeO_X$/$BiVO_4$‖CIGS/CdS/Pt	W	Partially,	Buffer pH 6.8,	3.7	10 min,	[21],

		0.1 cm^2	1 Sun		20% degradation	2018
Ni/TiO_2/$GaInP_2$/GaAs/NiMo	M	Yes, 1 cm^2	1 M KOH, 1 Sun	8.6	4 h, 10% degradation	[22], 2015
IrO_x‖FTO/AU/CuSCN/Cu_2O/Ga_2O_3/TiO_2/RuO_x wired to a Pb-based perovskite	W	No, -	Buffer pH 5, 1 Sun	4.55	10 h, 9% degradation	[23], 2020
$NiFeO_x$/H, Mo:$BiVO_4$/FTO‖FTO/AU/Sb_2Se_3/CdS/TiO_2/Pt	W	Partially, 0.32 cm^2	Buffer pH 7, 1 Sun	1.5	10 h, 25%	[24], 2020
$NiFeO_x$/Mo:$BiVO_4$‖Glass/Au/Cu_2O/Ga_2O_3/TiO_2/RuO_x	W	Partially, -	Buffer pH 9, 1 Sun	3	12h	[25], 2018
CoO_x-NiO_x/$BiVO_4$‖Ti-foil/CIGS/ZnSe/Pt series connected anode and cathode	M	Partially, Photoanode: 0.88 cm^2 Photocathode: 0.33 cm^2	Buffer pH 9.5, 1 Sun	1.1	20 h, 28% degradation	[26], 2017
FeNiOOH/Fe_2TiO_5/Fe_2O_3/ITO‖a-Si:H/μc-Si:H/ μc-Si:H/Ni foam	M	Yes, 1 cm^2	NaOH, 3 Suns	1.75	>24 h	[27], 2019
IrO_2(DSA)‖ITO/ETM/Perovskite/MoO_x/HTM/IZO/Au-pnn-Si/Ti/Pt	W	No, 0.3 cm^2	1 M H_2SO_4, 1 Sun	17.4	0.11 h	[28], 2020

Supplementary Note 1

XPS analysis of the pre- and post-operation BGLC587

Figure S7 shows XPS spectra for BGLC587 pre- and post-operation. The pre-operation survey spectrum shows the presence of Gd, La, Co and Ba together with C and O, while the post-operation survey is dominated by Na, F, O and C (**Figure S7a**). The La 3d and O 1s spectra pre-operation (**Figure S7b** and **c**) are similar to spectra reported for the $LaCoO_3$ phase.[29] The I_O component of O 1s can unambiguously be assigned to perovskite lattice oxygen, e.g., $LaCoO_3$, while the II_O component can be assigned to other oxide phases, under-coordinated oxygen and/or adsorbed O_2 or OH^- species.[30, 31] The Gd 4d spectrum (**Figure S7e**), which contains multiple components due to final state interactions with the $4f_7$ valence band electrons, was fitted by the procedure used by Thiede *et al*.[32] Although only one set of Gd components was necessary to obtain a good fit, the presence of more than one Gd phase cannot be completely ruled out.

The Ba 4d spectrum **Figure S7f** shows clear indications of two different Ba 4d states. This was also seen in the Ba 3d spectrum (**Figure S7d**), where the two components labelled I_{Ba} and II_{Ba} are consistent with previous reports for similar materials.[30, 31] The fact that the I_{Ba} and II_{Ba} components in the Ba $3d_{5/2}$ region are repeated with 2/3 of the peak area in the Ba 3d3/2 region verifies the identification as Ba 3d components.

The Co 2p spectrum is complicated, as the main peak is accompanied by several plasmon loss peaks, which vary in separation and intensity depending on the chemical state of Co.[33] To obtain a good fit of the spectrum in **Figure S7d**, the two components I_{Co} and II_{Co} had to be added. These components are repeated with 1/2 of the peak area in the Co $2p_{1/2}$ region, demonstrating the presence of both Ba and Co in the sample. Due to the overlap between Ba 3d and Co 2p, however, a detailed description of the Co phase(s) was not possible. The Co 3p region is less described in the literature, but a doublet splitting of 1.1 eV is assumed.[34] The Co 3p spectrum in **Figure S7g** was fitted with 3p doublet components at a fixed distance of 1.1 eV. In addition, a second doublet set is added to the high BE region. As for Co 2p, it is unclear whether these secondary components are an indication of Co in a different state, or if it is just part of the satellite structure of the main component.

A complete determination of the composition of the post-operation powder based on the XPS spectra was challenging due to low sample amounts. However, certain observations can be made:

Post-operation, the Co 2p/Ba 3d region is dominated by a component that is repeated with a 2:1 ratio between the 3/2 and 1/2 level, indicative of Co and thus suggesting a loss of Ba. The Co 2p component corresponds with the position of the weakest Co component in the pristine powder (II_{Co}). The component labelled as I_{Ba} in pre-operation is still present, repeating with a 3:2 ratio between the 5/2 and 3/2 levels. In the Ba 4d spectrum, the I_{Ba} components were also present, while the II_{Ba} region appeared broader, possibly indicating a mix of several Ba phases. To estimate the relative content of Ba before and after cycling, quantification was done based on two regions not affected by peak overlap: Ba 4d and Co 3d, which confirms that the Ba content decreases relative to Co (**Table S3**). For the post-operation powder the La 3d and Gd 4d peaks overlapped with F KLL and Si 2s, which means that a reliable quantification of the relative content of Co, Ba, Gd and La could not be obtained.

The Gd 4d peak is broadened in a way that can be fitted by assuming Gd in two different chemical states, with identical multiplet splitting. Co 3p is also broadened, and can be fitted by increasing the intensity of the weak peak found in the pristine powder. This could be consistent with the relative change in peak intensity observed for Co 2p, but a more extensive XPS investigation, including pure reference samples of the different candidate compositions, is needed to fully understand the chemical decomposition of the BGLC system upon cycling.

Supplementary Note 2

Performance of the PV-PEC with IrO_2 as the anode electrode

The STH begins at 8.4% (**Figure S25**), which is lower than the expected efficiency of 9.3% according to the fitted *j*-*V* curve of the mini module and the *j*-*E* curves of IrO_2 and NiMo (**Figure S26a**). This discrepancy is again attributed to ohmic losses in the electrolyte, but possibly due to the series connected potentiostat. In this case the evaporation of the electrolyte reduces the photocurrent density, but unlike the ea-PV-PEC, the efficiency of the device was constantly decreasing. After 24 h of continuous operation the efficiency was reduced to approx. 7.5% (**Figure S25**). The loss is mainly attributed to dissolution of IrO_2 as preliminary monolithic devices retrieved their efficiency after IrO_2 is electrodeposited again (see **Figure S27**). We took this device for outdoor operation as well and as expected by the laboratory performance, as well as the *j*-*E* curves of the HER and OER catalysts, an STH fluctuating around 7% was achieved (**Figure S28**). It can be seen that the sunlight intensity was significantly fluctuating due to passing clouds during the first hour of operation. It is also interesting to notice that under light intensities of around 0.4 and 0.2 Suns, the STH was well above 8%, reaching even 9%. Fittings of the *j*-*V* curves of the mini-PV module under different light intensities (0.2 to 0.8 Suns) to the *j*-*E* curves of IrO_2 and NiMo further confirm the performance range of the PV-PEC device (**Figure S26b-e**). The discrepancies observed may be attributed to different performance of the solar cells under natural sunlight and laboratory emulation. Finally, the IrO_2-coated FTO shows over 75% of the transmittance in the visible region, where the EQE has an excellent response (**Figure S29**).

Supplementary Note 3

Synthesis of BGLC-based double perovskites by sol-gel citrate method

Precursors

- Barium(II)carbonate (Alfa Cesar 99.8%) – 197.35 g mol^{-1}
- Gd(III)nitrate hexahydrate (Sigma-Aldrich 99.9%) – 451.36 g mol^{-1}
- La(III)nitrate hexahydrate (Sigma-Aldrich 99.99%) – 433.01 g mol^{-1}
- Co(II)nitrate hexahydrate (Sigma-Aldrich ≥ 98%) – 291.03 g mol^{-1}
- Citric acid monohydrate, (Sigma-Aldrich ≥ 99.0%) – 210.14 g mol^{-1}
- Ammonia 28% (AnalaR NORMAPUR®)

Citric acid (CA) was dissolved in water in an 1 L beaker on a hotplate using a 1:1 ratio of CA and moles of cations. A stoichiometric amount of $BaCO_3$ was slowly added until fully dissolution. Subsequently, the nitrates were added and dissolved. The pH was adjusted with concentrated ammonia until the solution became clear – pH below 6-7 will make the solution bleak and precipitate.

The water was evaporated on the hotplate at 150 °C under continuous magnetic stirring until a very viscous gel was formed. At this point the beaker was covered with a watch glass and put in a ventilated heating cabinet at 250 °C to obtain a more homogeneous heat distribution. The beaker was left in the heating cabinet for 2 hours for the combustion reaction to complete.

The xerogel/ash obtained from the combustion was crushed thoroughly in a mortar inside a fume hood. The powder was put in a crucible and covered with perforated heat resistant alumina foil to prevent contamination of furnace. The powder was fired at 450 °C for 2 h with a ramp rate of 300 °C/h to burn off most of the organic compounds. When done, the powder was crushed thoroughly a second time in a mortar and calcined at of 1100 °C for 5 h with a ramp rate of 250 °C/h.

Supplementary references

1. Zhao, B.; Zhang, L.; Zhen, D.; Yoo, S.; Ding, Y.; Chen, D.; Chen, Y.; Zhang, Q.; Doyle, B.; Xiong, X.; Liu, M., A tailored double perovskite nanofiber catalyst enables ultrafast oxygen evolution. *Nature Communications* **2017,** *8* (1), 14586.
2. Lyons, M. E. G.; Floquet, S., Mechanism of oxygen reactions at porous oxide electrodes. Part 2—Oxygen evolution at RuO2, IrO2 and IrxRu1−xO2 electrodes in aqueous acid and alkaline solution. *Physical Chemistry Chemical Physics* **2011,** *13* (12), 5314-5335.
3. Matsumoto, Y.; Kurimoto, J.; Sato, E., Oxygen evolution on SrFeO3 electrode. *Journal of Electroanalytical Chemistry and Interfacial Electrochemistry* **1979,** *102* (1), 77-83.
4. Jung, J.-I.; Jeong, H. Y.; Kim, M. G.; Nam, G.; Park, J.; Cho, J., Fabrication of Ba0.5Sr0.5Co0.8Fe0.2O3–δ Catalysts with Enhanced Electrochemical Performance by Removing an Inherent Heterogeneous Surface Film Layer. *Advanced Materials* **2015,** *27* (2), 266-271.
5. Grimaud, A.; May, K. J.; Carlton, C. E.; Lee, Y.-L.; Risch, M.; Hong, W. T.; Zhou, J.; Shao-Horn, Y., Double perovskites as a family of highly active catalysts for oxygen evolution in alkaline solution. *Nature Communications* **2013,** *4* (1), 2439.
6. Hua, B.; Sun, Y.-F.; Li, M.; Yan, N.; Chen, J.; Zhang, Y.-Q.; Zeng, Y.; Shalchi Amirkhiz, B.; Luo, J.-L., Stabilizing Double Perovskite for Effective Bifunctional Oxygen Electrocatalysis in Alkaline Conditions. *Chemistry of Materials* **2017,** *29* (15), 6228-6237.
7. Zhu, Y.; Lin, Q.; Hu, Z.; Chen, Y.; Yin, Y.; Tahini, H. A.; Lin, H.-J.; Chen, C.-T.; Zhang, X.; Shao, Z.; Wang, H., Self-Assembled Ruddlesden–Popper/Perovskite Hybrid with Lattice-Oxygen Activation as a Superior Oxygen Evolution Electrocatalyst. *Small* **2020,** *16* (20), 2001204.
8. Sun, H.; Hu, B.; Guan, D.; Hu, Z.; Fei, L.; Li, M.; Peterson, V. K.; Lin, H.-J.; Chen, C.-T.; Ran, R.; Zhou, W.; Shao, Z., Bulk and Surface Properties Regulation of Single/Double Perovskites to Realize Enhanced Oxygen Evolution Reactivity. *ChemSusChem* **2020,** *13* (11), 3045-3052.
9. Andersen, H.; Xu, K.; Malyshkin, D.; Strandbakke, R.; Chatzitakis, A., A highly efficient electrocatalyst based on double perovskite cobaltites with immense intrinsic catalytic activity for water oxidation. *Chemical Communications* **2020,** *56* (7), 1030-1033.
10. Yagi, S.; Yamada, I.; Tsukasaki, H.; Seno, A.; Murakami, M.; Fujii, H.; Chen, H.; Umezawa, N.; Abe, H.; Nishiyama, N.; Mori, S., Covalency-reinforced oxygen evolution reaction catalyst. *Nature Communications* **2015,** *6* (1), 8249.
11. Forslund, R. P.; Hardin, W. G.; Rong, X.; Abakumov, A. M.; Filimonov, D.; Alexander, C. T.; Mefford, J. T.; Iyer, H.; Kolpak, A. M.; Johnston, K. P.; Stevenson, K. J., Exceptional electrocatalytic oxygen evolution via tunable charge transfer interactions in La0.5Sr1.5Ni1−xFexO4±δ Ruddlesden-Popper oxides. *Nature Communications* **2018,** *9* (1), 3150.
12. Pan, Y.; Xu, X.; Zhong, Y.; Ge, L.; Chen, Y.; Veder, J.-P. M.; Guan, D.; O'Hayre, R.; Li, M.; Wang, G.; Wang, H.; Zhou, W.; Shao, Z., Direct evidence of boosted oxygen evolution over perovskite by enhanced lattice oxygen participation. *Nature Communications* **2020,** *11* (1), 2002.
13. Khaselev, O.; Turner, J. A., A Monolithic Photovoltaic-Photoelectrochemical Device for Hydrogen Production via Water Splitting. *Science* **1998,** *280* (5362), 425.
14. Licht, S.; Wang, B.; Mukerji, S.; Soga, T.; Umeno, M.; Tributsch, H., Efficient Solar Water Splitting, Exemplified by RuO2-Catalyzed AlGaAs/Si Photoelectrolysis. *The Journal of Physical Chemistry B* **2000,** *104* (38), 8920-8924.
15. Han, L.; Abdi, F. F.; van de Krol, R.; Liu, R.; Huang, Z.; Lewerenz, H.-J.; Dam, B.; Zeman, M.; Smets, A. H. M., Efficient Water-Splitting Device Based on a Bismuth Vanadate Photoanode and Thin-Film Silicon Solar Cells. *ChemSusChem* **2014,** *7* (10), 2832-2838.
16. Walczak, K.; Chen, Y.; Karp, C.; Beeman, J. W.; Shaner, M.; Spurgeon, J.; Sharp, I. D.; Amashukeli, X.; West, W.; Jin, J.; Lewis, N. S.; Xiang, C., Modeling, Simulation, and Fabrication of a Fully Integrated, Acid-stable, Scalable Solar-Driven Water-Splitting System. *ChemSusChem* **2015,** *8* (3), 544-551.
17. Fujii, K.; Nakamura, S.; Sugiyama, M.; Watanabe, K.; Bagheri, B.; Nakano, Y., Characteristics of hydrogen generation from water splitting by polymer electrolyte electrochemical cell directly connected with concentrated photovoltaic cell. *International Journal of Hydrogen Energy* **2013,** *38* (34), 14424-14432.
18. Kelly, N. A.; Gibson, T. L., Design and characterization of a robust photoelectrochemical device to generate hydrogen using solar water splitting. *International Journal of Hydrogen Energy* **2006,** *31* (12), 1658-1673.
19. Jacobsson, T. J.; Fjällström, V.; Sahlberg, M.; Edoff, M.; Edvinsson, T., A monolithic device for solar water splitting based on series interconnected thin film absorbers reaching over 10% solar-to-hydrogen efficiency. *Energy & Environmental Science* **2013,** *6* (12), 3676-3683.

20. Luo, J.; Im, J.-H.; Mayer, M. T.; Schreier, M.; Nazeeruddin, M. K.; Park, N.-G.; Tilley, S. D.; Fan, H. J.; Grätzel, M., Water photolysis at 12.3% efficiency via perovskite photovoltaics and Earth-abundant catalysts. *Science* **2014,** *345* (6204), 1593.
21. Varadhan, P.; Fu, H.-C.; Kao, Y.-C.; Horng, R.-H.; He, J.-H., An efficient and stable photoelectrochemical system with 9% solar-to-hydrogen conversion efficiency via InGaP/GaAs double junction. *Nature Communications* **2019,** *10* (1), 5282.
22. Verlage, E.; Hu, S.; Liu, R.; Jones, R. J. R.; Sun, K.; Xiang, C.; Lewis, N. S.; Atwater, H. A., A monolithically integrated, intrinsically safe, 10% efficient, solar-driven water-splitting system based on active, stable earth-abundant electrocatalysts in conjunction with tandem III–V light absorbers protected by amorphous TiO2 films. *Energy & Environmental Science* **2015,** *8* (11), 3166-3172.
23. Pan, L.; Liu, Y.; Yao, L.; Dan, R.; Sivula, K.; Grätzel, M.; Hagfeldt, A., Cu2O photocathodes with band-tail states assisted hole transport for standalone solar water splitting. *Nature Communications* **2020,** *11* (1), 318.
24. Yang, W.; Kim, J. H.; Hutter, O. S.; Phillips, L. J.; Tan, J.; Park, J.; Lee, H.; Major, J. D.; Lee, J. S.; Moon, J., Benchmark performance of low-cost Sb2Se3 photocathodes for unassisted solar overall water splitting. *Nature Communications* **2020,** *11* (1), 861.
25. Pan, L.; Kim, J. H.; Mayer, M. T.; Son, M.-K.; Ummadisingu, A.; Lee, J. S.; Hagfeldt, A.; Luo, J.; Grätzel, M., Boosting the performance of Cu2O photocathodes for unassisted solar water splitting devices. *Nature Catalysis* **2018,** *1* (6), 412-420.
26. Higashi, T.; Kaneko, H.; Minegishi, T.; Kobayashi, H.; Zhong, M.; Kuang, Y.; Hisatomi, T.; Katayama, M.; Takata, T.; Nishiyama, H.; Yamada, T.; Domen, K., Overall water splitting by photoelectrochemical cells consisting of (ZnSe)0.85(CuIn0.7Ga0.3Se2)0.15 photocathodes and BiVO4 photoanodes. *Chemical Communications* **2017,** *53* (85), 11674-11677.
27. Urbain, F.; Tang, P.; Smirnov, V.; Welter, K.; Andreu, T.; Finger, F.; Arbiol, J.; Morante, J. R., Multilayered Hematite Nanowires with Thin-Film Silicon Photovoltaics in an All-Earth-Abundant Hybrid Tandem Device for Solar Water Splitting. *ChemSusChem* **2019,** *12* (7), 1428-1436.
28. Karuturi, S. K.; Shen, H.; Sharma, A.; Beck, F. J.; Varadhan, P.; Duong, T.; Narangari, P. R.; Zhang, D.; Wan, Y.; He, J.-H.; Tan, H. H.; Jagadish, C.; Catchpole, K., Over 17% Efficiency Stand-Alone Solar Water Splitting Enabled by Perovskite-Silicon Tandem Absorbers. *Advanced Energy Materials* **2020,** *10* (28), 2000772.
29. Zhao, X.; Yang, Q.; Cui, J., XPS study of surface absorbed oxygen of ABO3 mixed oxides. *Journal of Rare Earths* **2008,** *26* (4), 511-514.
30. Jung, J.-I.; Edwards, D. D., X-ray photoelectron study on Ba0.5Sr0.5CoxFe1−xO3−δ (BSCF: x = 0.2 and 0.8) ceramics annealed at different temperature and pO2. *Journal of Materials Science* **2011,** *46* (23), 7415-7422.
31. Xu, X.; Chen, Y.; Zhou, W.; Zhu, Z.; Su, C.; Liu, M.; Shao, Z., A Perovskite Electrocatalyst for Efficient Hydrogen Evolution Reaction. *Advanced Materials* **2016,** *28* (30), 6442-6448.
32. Thiede, T. B.; Krasnopolski, M.; Milanov, A. P.; de los Arcos, T.; Ney, A.; Becker, H.-W.; Rogalla, D.; Winter, J.; Devi, A.; Fischer, R. A., Evaluation of Homoleptic Guanidinate and Amidinate Complexes of Gadolinium and Dysprosium for MOCVD of Rare-Earth Nitride Thin Films. *Chemistry of Materials* **2011,** *23* (6), 1430-1440.
33. Biesinger, M. C.; Payne, B. P.; Grosvenor, A. P.; Lau, L. W. M.; Gerson, A. R.; Smart, R. S. C., Resolving surface chemical states in XPS analysis of first row transition metals, oxides and hydroxides: Cr, Mn, Fe, Co and Ni. *Applied Surface Science* **2011,** *257* (7), 2717-2730.
34. Lebugle, A.; Axelsson, U.; Nyholm, R.; Mårtensson, N., ExperimentalLandMCore Level Binding Energies for the Metals22Ti to30Zn. *Physica Scripta* **1981,** *23* (5A), 825-827.
